# Toxicity of Magnetic Nanoparticles in Medicine: Contributing Factors and Modern Assessment Methods

**DOI:** 10.3390/ijms26178586

**Published:** 2025-09-03

**Authors:** Julia Nowak-Jary, Beata Machnicka

**Affiliations:** Department of Biotechnology, Institute of Biological Sciences, University of Zielona Gora, Prof. Z. Szafrana 1, 65-516 Zielona Gora, Poland; b.machnicka@wnb.uz.zgora.pl

**Keywords:** magnetic iron oxide nanoparticles, nanotoxicity, nanomedicine, in vitro and in vivo toxicity evaluation, in ovo methods, 3D bioprinting, organoids, imaging cytometry

## Abstract

With the rapid evolution of nanotechnology, magnetic iron oxide nanoparticles (MNPs)—primarily Fe_3_O_4_ and γ-Fe_2_O_3_—have gained prominence in biomedicine. Their extensive specific surface area, tunable surface functionalities, and intrinsic magnetic characteristics render them highly versatile for diverse clinical applications, including tumor visualization through Magnetic Resonance Imaging (MRI), radiolabeling, targeted radiotherapy, hyperthermia, gene transfer, drug delivery, Magnetic Particle Imaging (MPI), magnetic blood filtration and theranostic strategies. Nevertheless, ensuring the biocompatibility and non-toxicity of these nanostructures remains a fundamental prerequisite for their medical implementation. Hence, it is essential to continuously refine our understanding of MNP-related toxicity and pursue comprehensive research on this front. This article consolidates up-to-date insights into the evaluation of MNPs’ toxicological profiles, emphasizing the influence of physicochemical properties such as morphology, surface modifications, and electrostatic characteristics, along with operational factors like dosage and administration routes. Traditional toxicity testing strategies, including in vitro assays as first-line screening tools, together with standard ex vivo and in vivo models, are discussed. Special attention is given to the emerging role of New Approach Methodologies (NAMs), such as organoid formation, 3D bioprinting, in ovo chicken embryo assays, and image cytometry. These techniques offer ethical, human-relevant, and informative alternatives to animal testing, supporting more predictive and translationally relevant toxicity assessment of MNPs. Taken together, the integration of conventional assays with innovative NAMs, alongside careful consideration of physicochemical and operational factors, is essential to translate the laboratory promise of MNPs into safe and clinically effective nanomedicines.

## 1. Introduction

Magnetic iron oxide nanoparticles (MNPs), including magnetite (Fe_3_O_4_) and maghemite (γ-Fe_2_O_3_), consist of magnetic domains with permanent magnetization and represent a unique class of nanostructures used in medicine. Their ferromagnetic properties distinguish them from other nanoparticles, such as polymeric, metallic (e.g., gold or silver), dendrimeric, or carbon-based nanostructures. In magnetic materials, individual atomic magnetic moments align under an external magnetic field, resulting in a net magnetization; in ferromagnetic materials, this alignment persists even after the field is removed, producing a permanent magnetic moment. By contrast, superparamagnetic nanoparticles—such as many ultrasmall iron oxide nanocrystals—exhibit strong magnetization only in the presence of an external magnetic field, with thermal fluctuations rapidly randomizing magnetic moments once the field is removed. The term magnetic is used more broadly to encompass both ferromagnetic and superparamagnetic behaviors, as well as other magnetic phenomena relevant to nanoscale systems.

MNPs can be effectively guided to specific sites in the body using an external magnetic field, making them valuable as targeted drug delivery systems [1,2]. In vivo magnetic drug targeting increases local nanoparticle and payload concentrations at the target while reducing systemic exposure; gradient strength, blood flow, and tissue depth critically determine capture efficiency [3,4]. Well-established applications of MNPs include magnetic resonance imaging (MRI) [5] and hyperthermia treatment [6]. As MRI contrast agents, iron-oxide MNPs primarily shorten T_2_/T_2_* (negative contrast), and—when properly engineered or at specific field strengths/sequences—can also provide T_1_-weighting; clinical and translational studies have leveraged ultra-small MNPs for vascular and cellular imaging [7]. In magnetic hyperthermia, exposure to an alternating magnetic field converts magnetic losses (Néel/Brownian relaxation) into heat, allowing focal thermic doses within tumors; clinical studies (e.g., recurrent glioblastoma) demonstrate feasibility and safety of intratumoral MNP instillation combined with radiotherapy [8]. Magnetic nanoparticles are also applied in gene therapy [9], radiolabelling, and internal radiotherapy [10]. Magnetofection uses field-guided MNP–nucleic acid or viral complexes to increase vector contact with target cells and enhance transduction/transfection while lowering required vector doses; although clinical translation is ongoing, in vivo studies identify formulation and coating chemistry as key determinants of efficacy [11]. Radiolabeled iron-oxide MNPs enable multimodal imaging (MRI–PET/SPECT) (PET—positron emission tomography, SPECT—single photon emission computed tomography) and targeted radionuclide therapy by chelating diagnostic isotopes (e.g., ^68^Ga, ^64^Cu, ^89^Zr) or therapeutic emitters (e.g., ^177^Lu), improving dosimetry and image-guided treatment planning [12]. Certain types of iron oxide nanoparticles have demonstrated effectiveness in treating microbial infections [13]. Exploiting their enzyme-mimetic (peroxidase-like) activity, iron-oxide nanozymes catalytically activate peroxide to generate reactive intermediates that disrupt biofilms and kill pathogens. An additional important application of magnetic nanoparticles is Magnetic Particle Imaging, a technique that enables cell tracking, blood pool imaging, and image-guided magnetic hyperthermia [14]. A distinctive feature of MPI is that the signal originates exclusively from engineered nanoparticle tracers, thereby driving the rational design of magnetic nanoparticles with precisely controlled size, morphology, composition, and surface chemistry to fulfill the demands of targeted applications. More recently, MNPs have emerged as promising tools in bone tissue engineering [15]. Incorporation of MNPs into scaffolds permits remote, non-invasive actuation to promote osteogenic differentiation (via mechanotransduction pathways) and to guide cell recruitment/organization, while also enabling magnetically triggered release of osteo-inductive cues. Reviews highlight improved vascularization and mineralization in vivo when magnetic cues are combined with MNP-functionalized biomaterials [16,17]. A novel application of magnetic nanoparticles is their use in extracorporeal haemofiltration devices designed to selectively remove cytokines [18]. An example is the “IL-6-Sieve” system (IL-6—interleukin 6), in which magnetic Anti-IL-6 Beads are introduced into an extracorporeal circuit and subsequently removed together with interleukin-6 by a filter operating within a magnetic field. Preclinical studies in animals and a first-in-human trial demonstrated no serious adverse events and stable vital parameters. These findings highlight the promising translational potential of this blood purification technology for the treatment of inflammatory diseases [18].

A critical feature of magnetic nanoparticles for medical applications is their intended bioactivity and biocompatibility. In the context of biomaterials and nanomaterials, bioactivity refers to the capacity of a substance to induce a specific biological response at the interface between the material and living tissue. Biocompatibility, in turn, denotes the ability of a material to function with an appropriate host response in a given application, being non-toxic, non-injurious, and free from adverse immunological effects. Properly engineered magnetite and maghemite nanoparticles are generally regarded as safe, as iron is a naturally occurring trace element in the human body [19]. MNPs taken up by endocytosis accumulate in late endosomes and lysosomes, where acidic pH and chelating metabolites dissolve the iron oxide core, generating Fe^3+^ and Fe^2+^ [20,21,22]. Lysosomal ferrireductases, such as STEAP3 (six-transmembrane epithelial antigen of prostate 3), reduce Fe^3+^ to Fe^2+^, which is exported to the cytosol by DMT1 (divalent metal transporter) or TRPML1/MCOLN1 (transient receptor potential mucolipin 1) [20,21,23]. Once in the LIP (labile iron pool), this iron—regardless of origin—enters canonical metabolic pathways: storage in ferritin via H-chain ferroxidase activity [23,24], mitochondrial import through mitoferrins for heme synthesis and Fe–S cluster assembly via ferrochelatase and ISC machinery (iron-sulfur cluster assembly machinery) [25,26,27,28,29], or export by ferroportin with extracellular oxidation to Fe^3+^ by multicopper ferroxidases [30,31]. Ferritin turnover via NCOA4 (nuclear receptor coactivator 4)-mediated ferritinophagy and integration of mitochondrial iron utilization with systemic BMP6–SMAD–hepcidin signaling (BMP6—bone morphogenetic protein 6) ensure iron supply matches metabolic demand [31,32,33].

Several iron oxide–based magnetic nanoparticle formulations have received clinical approval for human use as imaging contrast agents, hyperthermia mediators, or therapeutic iron supplements [34]. From a regulatory perspective, all clinically approved MNPs have undergone rigorous evaluation under relevant medicinal product or medical device frameworks, including preclinical toxicology, pharmacokinetics, and clinical safety trials, before marketing authorization by agencies such as the U.S. Food and Drug Administration (FDA) or the European Medicines Agency (EMA) [35,36]. GastroMark^®^ (also marketed as Lumirem^®^; ferumoxsil) is composed of non-stoichiometric magnetite (Fe_3_O_4_), with a mean particle diameter of approximately 0.4 µm, and is coated with a poly[N-(2-aminoethyl)-3-aminopropyl]siloxane layer to enhance gastrointestinal stability and reduce aggregation [37]. Feridex^®^ (ferumoxides injectable solution) consists of dextran (DEX)-coated, non-stoichiometric magnetite (Fe_3_O_4_), with a hydrodynamic diameter of 120–180 nm, and includes mannitol and citrate as excipients [38]. Feraheme^®^ (ferumoxytol) is a carbohydrate-coated, superparamagnetic iron oxide nanoparticle formulation containing non-stoichiometric magnetite (Fe_3_O_4_) with a hydrodynamic diameter of 17–31 nm; the coating is polyglucose sorbitol carboxymethylether, which provides high colloidal stability and prolonged circulation [39]. NanoTherm^®^, used for intratumoral magnetic hyperthermia, contains iron oxide nanoparticles most commonly reported as magnetite (Fe_3_O_4_) with hydrodynamic diameters in the tens of nanometers range; however, detailed clinical specifications for particle size distribution and surface coating have not been disclosed by the manufacturer, and available literature reports variations depending on production batch [40,41]. Post-approval, these formulations remain subject to ongoing pharmacovigilance and quality control monitoring, including reporting of adverse events, stability testing, and compliance with current Good Manufacturing Practices (cGMP), to ensure continued safety and efficacy throughout their commercial lifecycle [35,42]. Nevertheless, the potential toxicity of MNPs is influenced by multiple parameters, including core size, coating type, surface charge, dosage, exposure duration, and the specific cells or tissues exposed; therefore, comprehensive toxicity assessments are required for each newly developed nanoparticle formulation.

It is well established that iron oxide nanoparticles can induce oxidative stress, which is a significant contributor to cell apoptosis [43]. The surface coating of magnetic nanoparticles plays a vital role in mitigating this effect. Thus, appropriate surface functionalization of MNPs not only enhances their stability and provides sites for the attachment specific drugs and biomolecules but also minimizes or eliminates their potential toxicity [44]. This review summarizes the factors affecting MNP toxicity and outlines the methods used to evaluate their toxicity in vitro, ex vivo, in vivo, and in ovo.

## 2. Factors Affecting the Potential Toxicity of MNPs

The potential toxicity of magnetic iron oxide nanoparticles is determined by their physicochemical properties, including size, shape, surface chemistry, and surface charge. In addition, technical factors such as dosage, route of administration, duration of exposure, and the specific cell line used for analysis can significantly influence the outcomes of toxicity studies. As a result, predicting the effects of a specific type of nanoparticles on living organisms remains highly challenging. Nevertheless, several general principles have been established regarding the impact of nanoparticle size and surface charge on their toxicity. Notably, size plays a crucial role in determining the biodistribution of nanoparticles within the body [45]. It can therefore be inferred that organs and tissues with the highest levels of MNPs accumulation are particularly susceptible to their potential toxic effects. Furthermore, it is essential to consider each organ’s ability to effectively clear MNPs. Smaller MNPs tend to accumulate in cells to a greater extent than larger particles, potentially leading to enhanced toxicity at the cellular level. In terms of shape, spherical nanoparticles degrade more slowly due to their smaller surface area available for degradation compared to cubic MNPs [46]. Additionally, positively charged nanoparticles are generally more toxic than their negatively charged counterparts, owing to their stronger interactions with cellular membranes.

**Figure 1:**
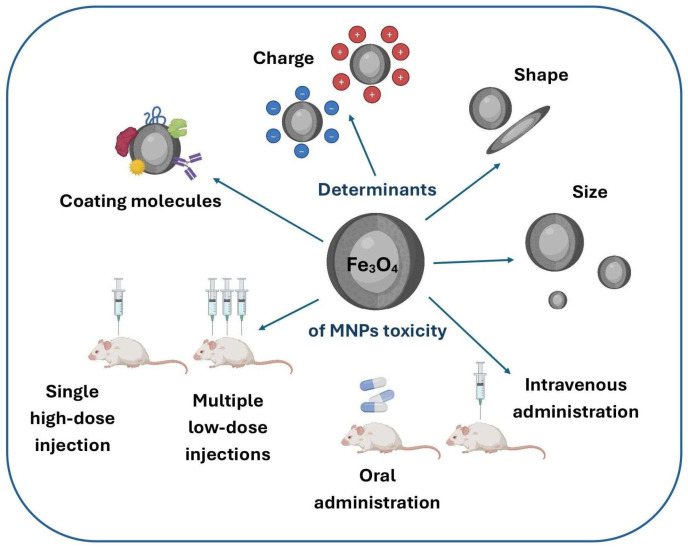
The main factors influencing the toxicity of MNPs. Several physicochemical and experimental determinants govern the biological effects of magnetic nanoparticles, including particle size, shape, surface charge, and coating molecules, as well as the route and regimen of administration. These parameters can significantly modulate cellular uptake, biodistribution, and the extent of toxic responses, thereby determining the overall safety profile of MNPs in biomedical applications. Created in Biorender. Julia Nowak-Jary. (2025) https://BioRender.com/ (accessed on 29 July 2025).

The main factors influencing the toxicity of MNPs are illustrated in Figure 1.

### 2.1. Size and Shape

Numerous reports have compared the in vivo and in vitro toxicity of MNPs based on their size. Within PEG (polyethylene glycol)-coated Fe_3_O_4_ spheres, Yang et al. systematically compared 10, 20, 30, and 40 nm MNPs in mice and found no size-dependent shifts in seven standard hepatic markers (ALT—alanine aminotransferase, AST—aspartate aminotransferase, TP—total protein, ALB—albumin, GLB—globulin, A/G—albumin/globulin ratio, GGT—gamma-glutamyl transferase) versus controls, but 10 nm particles increased TBIL/DBIL (total bilirubin/direct bilirubin) and lowered ALP (alkaline phosphatase); smaller particles also persisted longer in blood and modulated WBC/PMN (white blood cell count/polymorphonuclear leukocytes) without overt toxicity, alongside size-graded hepatic gene-expression changes related to oxidative stress, immune response, iron transport, metabolism, and apoptosis [47]. In rats receiving *N*-(2-aminoethyl)-3-aminopropyl trimethoxysilane (AEAPS)-coated Fe_3_O_4_ (10/20/40 nm), Li et al. noted size-associated differences in select serum biochemistry, with partial negative and positive correlations for LDH (lactate dehydrogenase) and urea, respectively, versus particle size [48]. For BSA (bovine serum albumin)-coated Fe_3_O_4_ (40 vs. 80 nm) and their PEGylated derivatives, Abakumov et al. attributed toxicity primarily to surface chemistry, dose, and exposure duration; diameter per se did not change most cytotoxic readouts (except proliferation) in fibroblasts and U251 cells [44]. Concordantly, some reports support greater toxicity of ultrasmall Fe_3_O_4_ (2.3–4.2 nm) at high intravenous doses (100 mg/kg) in mice, plausibly via oxidative-stress mechanisms, whereas 9.3 nm produced no apparent acute toxicity under the same conditions [49]. Conversely, in hepatoma lines (SK-Hep-1, Hep3B) 6 nm Fe_3_O_4_ were least cytotoxic, 9 nm induced mitochondrial ROS (reactive oxygen species)-dependent necrosis, and 14 nm compromised plasma-membrane integrity (LDH release), underscoring that size-toxicity relationships can invert across models/endpoints even within one core chemistry [50].

Using SMART (somatic mutation and recombination test) in *Drosophila*, Kaygisiz & Ciğerci observed that Fe_2_O_3_ < 100 nm were non-genotoxic across tested concentrations, whereas <50 nm Fe_2_O_3_ exhibited genotoxicity at 1 and 10 mM, indicating core-chemistry- and size-dependent effects that should not be conflated with Fe_3_O_4_ datasets [51].

With constant iron-oxide chemistry, rod-shaped particles showed higher cellular uptake than spheres—likely due to increased membrane contact area—and induced necrosis in non-tumorigenic cells at matched iron doses [52]. DSPE (1,2-distearoyl-sn-glycero-3-phosphoethanolamine)-PEG-coated nanocubes exhibited low cytotoxicity up to 0.5 mg Fe/mL in a murine monocyte–macrophage line [53], while hyaluronan-conjugated nanoworms elicited lower inflammatory responses than spherical HA-coated counterparts in an atherosclerosis-imaging context [54]. Across fibroblast assays, comparative exposure to spheres, nanoworms, rods, and polymeric magnetic beads produced abnormal cell morphology (e.g., shrinkage), with toxicity ranking beads < nanoworms < nanospheres at equivalent concentrations, highlighting morphology as an independent determinant of hazard [55].

Currently, the available data regarding cellular uptake mechanisms, cytotoxicity, biodistribution, and degradation of high-aspect-ratio magnetic nanomaterials remain limited compared to those of spherical nanoparticles. Consequently, generalizing and predicting the toxicological effects of such nanomaterials, particularly in the context of their degradation, remains challenging.

Comprehensive data on MNPs toxicity as related to size and shape are summarized in Table 1.

### 2.2. Surface Chemistry and Charge

Uncoated iron oxide nanoparticles are generally considered more toxic due to their propensity to release iron ions and induce oxidative stress. Surface modification of these nanoparticles with polymers, such as PEG, can effectively reduce their cytotoxic potential. Moreover, specific functional groups on the nanoparticle surface play a crucial role in determining surface charge, which in turn influences nanoparticle toxicity.

Within cells, the initial processing of exogenous material occurs in endosomes, where vacuolar H^+^-ATPases acidify the lumen to approximately pH 5.5 by actively pumping protons across the endosomal membrane [56]. The experiments conducted by Turina et al. demonstrated a continuous release of iron ions from bare (uncoated) MNPs, reaching 15.3% after 72 h in an acidic solution [57]. In comparison, MNPs coated with a thick layer of polylactide-co-glycolide (PLGA) released 9.56% of iron ions over the same period. Notably, nanoparticles coated with DEX and poly (vinyl alcohol) (PVA) dissolved even more rapidly than bare MNPs, with iron ion release levels reaching 20.4% and 21.9%, respectively. Rabel et al. provided an explanation for this phenomenon, suggesting that protonation of DEX- and PVA-coated MNPs under acidic conditions increases their affinity for water and dissolution agents, leading to accelerated iron ion release [58].

Colloidal behavior can confound apparent “toxicity rankings”: because aggregation and agglomeration depend on surface charge, coating, and medium composition, they modulate the delivered cellular dose and particle–cell contact [59]. Illustratively, Závišová et al. examined A549 lung adenocarcinoma cells exposed to 10 nm iron-oxide cores functionalized with PEG, bovine serum albumin, or PEG–PLGA [60]. All formulations produced a concentration-dependent decrease in viability. After 24 h, PEG-PLGA–coated MNPs caused the most pronounced growth inhibition, whereas uncoated particles were less cytotoxic. The authors attributed the higher cytotoxicity of PEG-PLGA–coated MNPs primarily to their large hydrodynamic diameter (Dh ≈ 155 nm), which promoted colloidal instability and slow sedimentation onto the culture substrate, likely forming a film over adherent cells that imposed mechanical stress, impeded nutrient exchange, and perturbed cell–cell communication. By contrast, bare MNPs (Dh ≈ 44 nm) were at least twofold less cytotoxic than PEG- and PEG-PLGA–coated counterparts (Dh ≈ 76 nm and ≈ 155 nm, respectively). Overall, these findings indicate that hydrodynamic size and sedimentation-driven colloidal behavior exerted a stronger influence on the apparent cytotoxicity than the nominal surface chemistry.

In another study, superparamagnetic Fe_3_O_4_ cores (10 nm) were functionalized with three biopolymers—cationic hydroxyethyl cellulose (HEC), anionic nanocrystalline cellulose (NCC), and the non-ionic synthetic polymer poly (vinylpyrrolidone) (PVP)—and their cytotoxicity was assessed in human fetal osteoblast (hFOB) cells [61]. Uncoated nanoparticles showed the greatest cytotoxicity, whereas all coatings significantly attenuated toxicity. Among the coated formulations, NCC-modified particles were the most cytotoxic. Notably, HEC-coated Fe_3_O_4_ displayed lower-than-expected cytotoxicity despite the typical strong membrane affinity of cationic surfaces. The authors attributed the higher toxicity of NCC-coated particles to incomplete surface coverage, leaving regions of the iron-oxide core exposed, whereas both HEC and PVP achieved full coverage. When directly compared, PVP-coated nanoparticles produced a modestly greater improvement in cell viability than HEC-coated counterparts.

The following cases further demonstrate that differences in coating chemistry drive the observed biological responses to MNPs. RNA-seq profiling in adult zebrafish (*Danio rerio*) exposed for 7 days to bare or starch-stabilized MNPs showed significant, tissue-wide transcriptomic remodeling in both gill and liver; uncoated particles exerted stronger effects in gills, whereas starch-coated particles produced more pronounced hepatic perturbations, with pathway analyses indicating overlapping regulatory networks [62].

Genotoxicity batteries likewise revealed formulation dependence: PEG-coated MNPs were mutagenic in the Ames assay but non-clastogenic in mammalian tests, whereas PEI (polyethylenimine)-coated MNPs were non-genotoxic across all three standard assays [63]. At a complementary biophysical level, combined simulation and experiment with DPPC (1,2-dipalmitoyl-sn-glycero-3-phosphocholine) bilayers indicated that PVA- and poly (arabic acid)–coated magnetite neither penetrated the hydrocarbon core nor destabilized the bilayer, consistent with benign early-stage interactions [64].

Consistent with the central role of surface chemistry and interfacial charge, Yang et al. synthesized Fe_3_O_4_ MNPs bearing hydroxyl (TEOS—tetraethyl orthosilicate), amine (APTMS—(3-aminopropyl)trimethoxysilane), or mixed TEOS/APTMS functionalities and profiled responses in normal fibroblasts and fibrosarcoma cells [65]. Positively charged APTMS-coated particles caused a more pronounced loss of viability at concentrations > 600 µg/mL than other variants; LDH release was broadly comparable at matched doses, except that APTMS > 800 µg/mL yielded the highest LDH. SEM/TEM (scanning electron microscopy/transmission electron microscopy) showed that negatively charged bare and TEOS-coated MNPs adhered with localized aggregation, whereas positively charged APTMS and TEOS/APTMS formed stronger membrane attachments—consistent with electrostatic attraction to the negative resting potential of cell membranes—and, in genotoxicity assays, APTMS and TEOS/APTMS induced dose-dependent DNA damage in normal cells, whereas bare and TEOS-coated MNPs did not. Coating-dependent immunological and endothelial readouts further support these trends: PVA-coated MNPs reduced antigen processing and CD4^+^ T-cell stimulation in human monocyte-derived dendritic cells [66], while DEX- and PEG-coated iron-oxide MNPs were less cytotoxic toward aortic endothelial cells than uncoated controls [65]. In another study, charge effects were shown to generalize across materials: amine-functionalized (positively charged) silicon nanoparticles elicited greater cytotoxicity—manifested as reduced mitochondrial activity and impaired phagocytosis—than neutral azide-functionalized analogs, whereas carboxylated (negatively charged) particles were only minimally cytotoxic [67].

The breadth of available functionalization chemistries makes it difficult to predict specific biological responses a priori, and nanoparticle size often modulates the apparent impact of a given surface coating. The relationship between magnetic nanoparticle surface charge and toxicity also remains unresolved: while positively charged particles are generally expected to be more cytotoxic due to stronger membrane interactions, individual studies report conflicting outcomes, underscoring that charge–toxicity effects are context dependent and not fully understood. Taken together, current evidence indicates that bare iron-oxide MNPs tend to pose a higher intrinsic hazard via acid-promoted iron release and oxidative stress, whereas functionalized MNPs can either attenuate or exacerbate apparent toxicity depending on how the coating tunes surface charge, dissolution kinetics, colloidal stability, and cell–membrane interactions. Because aggregation and hydrodynamic size can dominate assay readouts, size- and surface-dependent effects should be interpreted within a single, well-defined MNPs class under matched media and exposure conditions, rather than compared across heterogeneous formulations [57,59,60].

### 2.3. Technical Aspects

When evaluating the toxicity of magnetic nanoparticles, it is essential to consider both dosage and treatment duration. It is well established that higher doses of nanoparticles are likely to result in increased toxicity, and numerous studies have addressed this relationship. For instance, Gokduman et al. investigated the concentration-dependent (0–400 μg/mL) and exposure-dependent (single dose vs. cumulative dose) effects of bare superparamagnetic iron oxide nanoparticles (Fe_3_O_4_, 10 nm) on primary rat hepatocytes over varying time periods [68]. The data revealed that hepatocyte viability decreased as MNP concentration increased, regardless of whether the treatment was administered as a single dose or cumulatively. The study also showed the damaging effects of MNPs on primary rat hepatocytes progressed with time. Specifically, after 48 h, approximately 65% of hepatocytes exposed to 400 μg/mL MNPs had died in both treatment groups. By the seventh day, nearly all hepatocytes treated with 400 μg/mL nanoparticles were non-viable in both groups. Moreover, no statistically significant differences in cell viability or LD_50_ values were observed between the single-dose and cumulative-dose groups at either 24- or 48 h post-treatment. However, substantial differences in hepatocyte-specific functions—such as albumin and urea synthesis—were evident between the two groups after 48 h, whereas differences at the 24 h mark were not statistically significant.

In a separate study, Gong et al. evaluated the size-, concentration-, and time-dependent toxicity of superparamagnetic core/shell GoldMag nanoparticles (GMNPs with Fe_3_O_4_ core) in human umbilical vein endothelial cells (HUVECs) [69]. The study demonstarted key cellular behaviors—including proliferation, cytoskeletal organization, migration, tube formation, apoptosis, and ROS generation—were dependent on both GMNP concentration and exposure duration. No significant nanotoxicity was observed in HUVECs exposed to 50 nm GMNPs at concentrations up to 25 μg/mL for 12 h, or to 30 nm GMNPs at concentrations up to 50 μg/mL for 24 h. However, exposure to larger GMNPs at higher concentrations and longer durations led to increased ROS production in HUVECs.

Palacios-Hernandez et al. investigated the cytotoxicity and apoptotic responses of human coronary artery endothelial cells exposed to PVP-coated MNPs [70]. The cells were treated with various concentrations (0, 25, 50, 100, and 200 μg/mL) of MNPs. The results revealed time- and concentration-dependent cytotoxicity following exposure to 20 nm PVP-coated MNPs for periods ranging from 3–6 h up to 24 h, as assessed by Alamar Blue and RT-CES (real-time cell elactronic sensing) assays. In another study, PEG—PLGA-coated Fe_3_O_4_ nanoparticles were shown to exhibit minimal cytotoxicity toward cancer cells and normal fibroblasts at concentrations up to 100 μg/mL. However, high-dose exposure induced autophagy-dependent ferroptosis [71].

The route of nanoparticle administration plays a crucial role in determining the organs most susceptible to toxicity. For instance, pulmonary administration of bare Fe_2_O_3_ nanoparticles in Wistar rats has been shown to induce lung inflammation [72]. In addition, nanoparticles of various types can be efficiently absorbed through the gastrointestinal tract, either directly or via secondary ingestion of inhaled particles [73].

Oral administration of low doses of magnetic nanoparticles is generally considered safe, with only mild side effects such as nausea, vomiting, or flatulence reported for various types of Fe_3_O_4_ NPs [74]. No observable pathological changes in the stomach or other digestive organs have been detected, even at high doses up to 200 mg/kg [75]. However, Babadi et al. reported that oral administration of bare magnetite NPs may result in hormonal imbalances [76].

Intravenous injection of MNPs has been associated with several adverse effects, including oxidative stress and DNA damage in the heart (bare Fe_3_O_4_ nanoparticles) [77], necrosis of cardiac muscle tissue (PEGylated solid lipid NPs) [78], hemolysis, elevated AST and ALT levels (Fe_3_O_4_ NPs with different sizes and coatings) [79], and apoptosis in human skin fibroblasts (bare and Ag-coated Fe_3_O_4_ NPs) [80].

Toxicity of magnetic nanoparticles is strongly dependent on dose, particle size, coating, exposure duration, and route of administration. In vitro studies have consistently shown concentration- and time-dependent cytotoxicity, with higher doses leading to reduced cell viability, increased ROS production, and alterations in hepatocyte- or endothelial-specific functions. Surface modifications such as PEG or PLGA coatings can mitigate toxicity at lower concentrations, although high-dose exposure may still induce autophagy or ferroptosis. In vivo, oral administration of MNPs at low doses appears relatively safe, whereas intravenous or pulmonary delivery has been associated with inflammation, oxidative stress, DNA damage, and organ-specific toxic effects.

To clarify the sources of variability in reported MNPs toxicity, the determinants were categorized into three domains—MNP-intrinsic, experimental model-dependent, and technical (Table 2).

## 3. Primary Techniques for the Assessment of the Toxicity of MNPs

**Figure 2:**
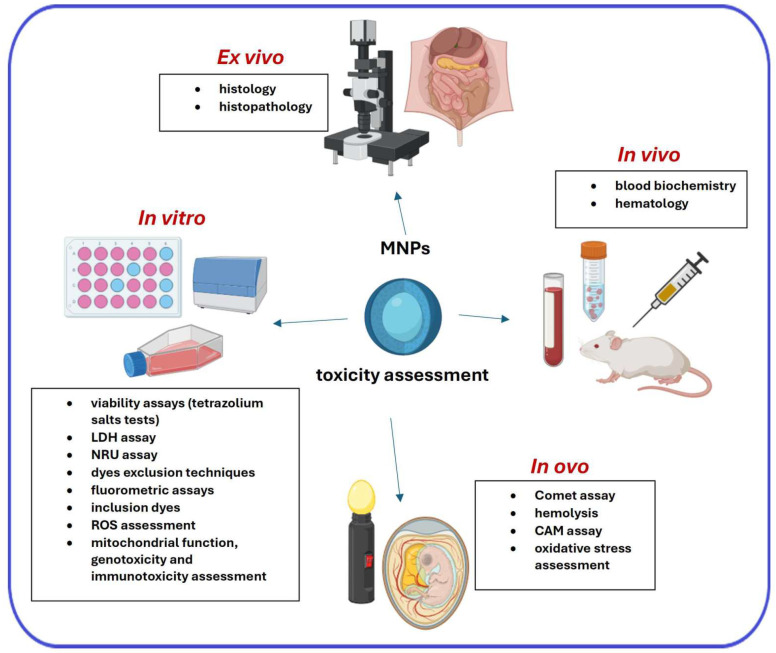
Methods and representative assays used for the evaluation of MNPs toxicity. Toxicological assessment of magnetic nanoparticles relies on a broad spectrum of complementary strategies, including in vitro assays (e.g., tetrazolium-based viability assays, LDH release, NRU (neutral red uptake assay), ROS measurement, genotoxicity and immunotoxicity tests), ex vivo techniques (histology and histopathology), in vivo models (blood biochemistry and hematological analysis), as well as in ovo approaches (e.g., CAM assay, comet assay, hemolysis, oxidative stress evaluation). Together, these methods provide a comprehensive view of MNP-induced biological responses at cellular, tissue, and organismal levels. Created in Biorender. Julia Nowak-Jary. (2025) https://BioRender.com/ (accessed on 29 July 2025).

The assessment of nanoparticle toxicity encompasses four primary categories: in vitro, ex vivo, in vivo and in ovo (Figure 2). In vitro assays typically entail the examination of individual isolated cells under specific and controlled laboratory conditions. Ex vivo studies involve the extraction of organs, tissues, or cells from a living organism, followed by testing outside the organism to monitor physiological responses in real time. In vivo assessments evaluate the intricate interactions among various biological systems (cardiovascular, respiratory, endocrine, digestive, renal, and nervous) within living organisms. Conversely, in ovo studies utilize primarily chicken embryonic model for toxicity evaluation. However, as in ovo methods can be successfully classified as part of the New Approach Methodologies (NAMs), they have been described in detail in Section 4.

### 3.1. In Vitro Techniques

#### 3.1.1. Cells Proliferation/Viability Investigation

The primary objective in evaluating the toxicity of nanoparticles is to assess their effects on cell proliferation and viability. Colorimetric assays based on tetrazolium salts are widely used for this purpose. These salts act as substrates for cellular dehydrogenases and reductases, and in the presence of NADH/NADPH (nicotinamide adenine dinucleotide/nicotinamide adenine dinucleotide phosphate), they are reduced to formazan products, producing distinct color changes. These reactions occur exclusively in metabolically active cells that can generate NAD(P)H. Consequently, tetrazolium-based assays are commonly employed to distinguish between viable, metabolically active cells and dead cells.

Among the most frequently used tetrazolium-based assays are the MTT (3-(4,5-dimethylthiazol-2-yl)-2,5-diphenyltetrazolium bromide), XTT (2,3-bis-(2-methoxy-4-nitro-5-sulfophenyl)-2H-tetrazolium-5-carboxanilide), MTS (3-(4,5-dimethylthiazol-2-yl)-5-(3-carboxymethoxyphenyl)-2-(4-sulfophenyl)-2H-tetrazolium), and WST (2-(4-iodophenyl)-3-(4-nitrophenyl)-5-(2,4-disulfophenyl)-2H-tetrazolium) assays [83].

The MTT assay, which remains the most widely applied method, relies on the reduction of MTT to a violet-blue, water-insoluble formazan product through cleavage of the tetrazole ring. This reaction occurs exclusively in viable, metabolically active cells. The resulting formazan crystals are then dissolved using a solvent, typically dimethyl sulfoxide (DMSO), and absorbance is measured, generally at 570 nm [84,85,86,87,88,89,90,91,92,93].

The XTT assay involves the bioreduction of XTT to a water-soluble, highly colored formazan product in viable cells, allowing for direct absorbance measurements using a multi-well plate reader (ELISA (enzyme-linked immunosorbent assay) reader).

The MTS assay similarly enables viable cells to generate soluble formazan products directly in the culture medium, thereby eliminating the need for a separate dissolution step. The MTS reagent can also be used with electron coupling reagents such as phenazine methosulfate (PMS) or phenazine ethosulfate (PES), which facilitate formazan production by enabling tetrazolium reduction within or on the surface of viable cells [94].

The WST assays use water-soluble tetrazolium salts, which yield formazan products with varying absorption spectra. WST-1 generates a highly water-soluble formazan via mitochondrial dehydrogenase activity, in the presence of an electron mediator such as 1-methoxy PMS. The amount of formazan produced is proportional to mitochondrial dehydrogenase activity, thus serving as a reliable indicator of metabolic activity. WST-1 offers sensitivity comparable to XTT, but with lower cytotoxicity, and it eliminates the need for a dissolution step—an advantage particularly valuable in high-throughput drug screening.

WST-8, a slightly yellow tetrazolium salt, is reduced by viable cells to form an orange-colored, water-soluble formazan product. The amount of formazan correlates linearly with the number of viable cells over a wide range (200–25,000 cells per well) and across various cell lines, including non-adherent cells. WST-8 has been reported to provide higher sensitivity in detecting cell viability compared to MTT, MTS, XTT, and WST-1 assays [83]. It is also noteworthy that a commercially available, ready-to-use assay, the Cell Counting Kit-8 (CCK-8), employs WST-8 for rapid and highly sensitive quantification of cell viability.

The assessment of cellular viability commonly employs the LDH assay [44,83]. LDH, an intracellular enzyme, is released into the culture medium following the loss of membrane integrity. Increased membrane permeability reflects cellular processes such as apoptosis, necrosis, or other forms of damage. LDH activity involves the oxidation of NADH to NAD^+^, coupled with the conversion of lactate to pyruvate. In the LDH assay, NADH also facilitates the reduction of the yellow tetrazolium salt INT (2-(4-iodophenyl)-3-(4-nitrophenyl)-5-phenyl-2H-tetrazolium, also known as iodonitrotetrazolium), to a water-soluble red formazan dye. The amount of formazan produced, quantified by measuring absorbance at 490 nm, directly correlates with the total LDH activity in the culture and, consequently, with the number of damaged cells [95].

The neutral red uptake (NRU) colorimetric assay is widely used to quantify viable cells in monolayer cultures. This assay relies on the ability of viable cells to incorporate and retain the dye neutral red (3-amino-7-dimethylamino-2-methylphenazine hydrochloride) within their lysosomes. The uptake of neutral red depends on the cell’s capacity to maintain intracellular pH gradients, which is closely linked to ATP production [96]. The bound dye is subsequently extracted from viable cells using an acidified ethanol solution, and its absorbance is measured by spectrophotometry.

Cell viability is commonly evaluated using dye exclusion assays, which are designed to distinguish viable cells from non-viable ones within a cell suspension. Among these, the trypan blue exclusion assay is widely used due to its simplicity and effectiveness [90]. In this assay, viable cells with intact plasma membranes exclude the dye, whereas non-viable cells with compromised membranes take up the dye and appear blue under microscopic examination.

Fluorometric assays provide an alternative to exclusion dyes and colorimetric methods. A notable example is the Alamar Blue (resazurin) assay, which is based on the enzymatic reduction of resazurin to resorufin by metabolically active cells. Resorufin is a pink, fluorescent compound that diffuses into the culture medium. The associated color change can be quantitatively assessed over a broad linear range, typically from 50 to 50,000 cells, using excitation wavelengths of 530–570 nm and emission wavelengths of 580–620 nm. The Alamar Blue assay is a sensitive, reliable, and straightforward method for evaluating cell viability and proliferation [97]. Intracellular inclusion dyes require both enzymatic activity within the cell and intact plasma membrane integrity. Cytoplasmic esterases metabolize non-fluorescent precursors into fluorescent derivatives. A representative inclusion dye is calcein acetoxymethyl ester (calcein-AM), a non-fluorescent, cell-permeant compound that is converted by intracellular esterases into calcein, an anionic fluorescent molecule [86]. Following staining, cells are typically analyzed using a flow cytometer, with excitation and emission wavelengths set at 488 nm and 520 nm, respectively [98]. Another fluorometric viability marker is 5-carboxyfluorescein diacetate acetoxymethyl ester (5-CFDA-AM). Similarly to Alamar Blue, 5-CFDA-AM targets nonspecific intracellular esterases present in viable cells. Upon enzymatic conversion, 5-CFDA-AM is transformed into carboxyfluorescein, a polar fluorescent compound that is impermeable to the plasma membrane in viable cells, thereby retaining the signal within the cytoplasm [44].

#### 3.1.2. Oxidative Stress Assessment

The generation of ROS is a key indicator of nanoparticle-induced cytotoxicity. A widely used method for quantifying ROS production is the DCFDA (2′,7′-dichlorofluorescin diacetate) Cellular ROS Assay Kit, commercially available from various vendors. This assay utilizes the cell-permeant reagent DCFDA (also referred to as H_2_DCFDA, DCFH-DA, or DCFH (2′,7′-dichlorodihydrofluorescin diacetate) to measure intracellular ROS levels in live cells. Once inside the cell, DCFDA is deacetylated by esterases to a non-fluorescent intermediate, which is subsequently oxidized by ROS to form 2′,7′-dichlorofluorescein, a highly fluorescent compound. Fluorescence is then detected using a spectrophotometer with excitation and emission wavelengths of 485 nm and 535 nm, respectively [44,99].

Alternative approaches for assessing ROS generation involve the quantification of antioxidant enzyme levels, including glutathione peroxidase (GPx), catalase, and superoxide dismutase (SOD) [100]. Superoxide dismutases catalyze the dismutation of superoxide radicals into hydrogen peroxide and molecular oxygen, while catalase and various peroxidases further convert hydrogen peroxide into water; catalase also produces molecular oxygen as a by-product. Within the glutathione system, glutathione reductase (GR) and glucose-6-phosphate dehydrogenase (G6PD) do not directly interact with ROS but play a crucial role in supporting the antioxidant function of GPx by maintaining intracellular levels of reduced glutathione [100].

Immunohistochemical analysis is a valuable technique for determining the cell-specific expression of antioxidant enzymes. Specific immunostaining protocols are available for both tissue sections and cultured cells to detect SOD, catalase, and GPx. Standard methodologies—such as immunohistochemistry, immunofluorescence, and immunogold labeling—are widely used in histological laboratories. Commercially available antibodies can be applied to both fresh and fixed tissues or cells [100].

The detection of highly reactive nitrogen species (RNS), particularly nitric oxide (NO), presents significant technical challenges due to NO’s rapid chemical reactivity with various biological molecules, including free radicals, metal ions, and thiol groups [85,101]. Several direct methods exist for NO quantification, such as electron paramagnetic resonance (EPR/ESR), electrochemical assays using NO-specific microelectrodes, and fluorescence-based assays. Fluorescence assays rely on the chemical interaction between NO (or its reactive intermediates) and a non-fluorescent probe, resulting in the formation of a fluorescent product. Indirect methods are also commonly employed, including the measurement of nitrate and nitrite levels. However, these methods are susceptible to interference from environmental contaminants—such as nitrate/nitrite residues in reagents, culture media, or plasticware—which can compromise the accuracy of the results [101].

It is well established that mitochondria are the primary sites of reactive oxygen species generation within the cell. Conditions characterized by antioxidant depletion and excessive ROS production are known to cause mitochondrial damage. Consequently, mitochondrial dysfunction represents a key hallmark of the early stages of programmed cell death. This dysfunction includes alterations in mitochondrial membrane potential, a critical indicator of mitochondrial health, as well as changes in the organelle’s redox state [102]. To detect ROS generated specifically within mitochondria, specialized mitochondria-targeted fluorescent probes are employed. One such probe is MitoSOX™ Red—mitochondria-targeted hydroethidine probe (also referred to as Mito-HE), a dihydroethidium derivative conjugated with a cationic triphenylphosphonium group. This positively charged moiety facilitates rapid accumulation of the probe within mitochondria, enabling the specific detection of superoxide anions and other ROS produced in these organelles. MitoSOX Red can be used in fluorometric assays, fluorescence microscopy, and flow cytometry for the quantitative and qualitative assessment of mitochondrial ROS production.

Reactive oxygen and nitrogen species compromise the integrity of the mitochondrial membrane, leading to the induction of mitochondrial permeability transition (MPT) through the opening of a non-specific pore known as the permeability transition (PT) pore. This pore allows the unregulated passage of solutes with a molecular weight below 1500 Da. As a result, mitochondria undergo depolarization, the uncoupling of oxidative phosphorylation, and pronounced swelling. Disruption of the mitochondrial membrane potential (MMP) is a critical intracellular event that follows the initiation of apoptosis. MMP can be assessed by monitoring the uptake of rhodamine 123, a lipophilic, cationic fluorescent dye [103]. Rhodamine 123 preferentially accumulates in active mitochondria with intact membrane potential, where it localizes to the inner mitochondrial membrane and emits strong green fluorescence. A reduction or absence of fluorescence indicates mitochondrial dysfunction or depolarization.

#### 3.1.3. Genotoxicity Evaluation

The potential genotoxicity of nanoparticles (NPs) represents a significant risk factor contributing to cell death. Consequently, numerous studies have investigated the impact of MNPs on genomic integrity. A widely used technique for detecting DNA damage in eukaryotic cells is the comet assay, also known as single-cell gel electrophoresis (SCGE) [104]. The comet assay quantitatively measures the extent of DNA fragmentation based on the migration of denatured DNA from the nucleus during electrophoresis. This method offers several advantages over other genotoxicity assays, including its ability to detect DNA damage at the single-cell level, its high sensitivity, low sample size requirements, simplicity, and cost-effectiveness. Moreover, the comet assay can be adapted to detect a variety of DNA lesions and is highly flexible under diverse experimental conditions. The underlying principle of the comet assay involves the migration of negatively charged low molecular weight DNA fragments—indicative of strand breaks—toward the anode under an electric field. The procedure begins with the lysis of cells using a hypertonic, non-ionic detergent solution, which removes the plasma membrane, cytoplasm, and nuclear proteins, leaving behind nucleoids containing supercoiled DNA. During electrophoresis, damaged DNA migrates from the nucleoid, forming a characteristic “comet tail.” The extent of DNA damage is quantified based on the length and intensity of this tail, with longer tails indicating greater DNA fragmentation.

Various methodologies utilize specific biomarkers to assess the impact of nanomaterial exposure on genetic material. Among these, a frequently studied biomarker of oxidative DNA damage is 8-hydroxy-2′-deoxyguanosine (8-OHdG) [105]. 8-OHdG has been extensively investigated due to its association with nucleobase mutations, particularly CG to AT transversions. Owing to guanine’s lower oxidation potential relative to other DNA bases, it is highly prone to oxidative modifications and is considered a prominent marker of oxidative stress. The ELISA is commonly employed to detect 8-OHdG in biological samples. ELISA offers several advantages, including rapid analysis, cost-effectiveness, and low sample volume requirements. However, a known limitation of this technique is the potential cross-reactivity of antibodies with guanine or structurally related compounds. In addition to immunoassays, chromatographic methods such as high-performance liquid chromatography (HPLC) are also widely used for the analysis of oxidative DNA lesions.

Genetic damage can also be detected using various fluorescent dyes. Propidium iodide (PI) binds to DNA and RNA in non-viable cells or cells with irreversibly damaged membranes, making it a useful indicator of cell death and membrane integrity loss [106]. Hoechst dyes, such as bisbenzimide trihydrochloride, selectively bind to the minor groove of double-stranded DNA—preferentially to A/T-rich regions—resulting in enhanced fluorescence intensity [107].

Assessment of DNA fragmentation can also be conducted using the deoxynucleotidyl transferase dUTP nick end labeling (TUNEL) assay, which enables the precise detection of both single- and double-stranded DNA breaks through the enzymatic incorporation of modified nucleotides at the sites of damage [108]. Additionally, the metabolic incorporation of tritiated thymidine (^3^H-thymidine) into cellular DNA indicate cell cycle arrest, apoptosis, and DNA damage, and thus serve as another marker of genotoxic stress [109].

#### 3.1.4. Apoptosis Detection

The initiation and regulation of apoptosis involve a complex network of molecular events, prominently featuring the caspase family of cysteine proteases. Among them, caspase-3 is recognized as a key executioner enzyme in the apoptotic cascade. Upon activation, caspases cleave various cellular substrates, including cytoskeletal components and proteins involved in RNA splicing and DNA repair. Therefore, measuring caspase-3 activity serves as a reliable indicator of experimental treatments, including exposure to nanoparticles. Caspase activity is commonly assessed using profluorescent tetrapeptide substrates [110]. In their uncleaved form, these substrates exhibit minimal fluorescence due to poor excitation and emission properties. Upon cleavage by active caspase-3, the fluorophore is released from the peptide backbone, resulting in a quantifiable fluorescence signal that correlates with enzymatic activity.

Cells undergoing apoptosis can be distinguished using acridine orange/ethidium bromide (AO/EB) staining. This method is based on the differential permeability of cell membranes: acridine orange (AO) permeates all cells and binds to DNA, emitting green fluorescence, whereas ethidium bromide (EB) penetrates only cells with compromised membranes—a hallmark of apoptosis or necrosis—and intercalates with DNA to produce red fluorescence [84]. Thus, viable cells exhibit green fluorescence, while apoptotic or necrotic cells display orange to red fluorescence depending on the degree of membrane disruption.

#### 3.1.5. Immunotoxicity Assessment

The lymphocyte proliferation assay (LPA) is widely used to evaluate immunotoxicity [111]. LPA assesses the capacity of lymphocytes cultured in short-term tissue culture to undergo clonal expansion in response to stimulation with a foreign molecule, antigen, or mitogen under in vitro conditions. In addition to LPA, several established techniques are employed for in vitro immunotoxicity assessment. These include ELISA, quantitative reverse transcription polymerase chain reaction (qRT-PCR), flow cytometry, transcriptomics, multiplex immunoassays, and immune repertoire sequencing. Such methods are used, for example, to quantify antibody titers and to characterize both cellular and humoral immune responses [111].

#### 3.1.6. Membrane Function Detection

Nanoparticle-induced cytotoxicity in exposed cells can be assessed by evaluating the functional integrity of the plasma membrane, particularly its ability to sustain transmembrane ion transport. One effective approach involves measuring the activity of membrane-bound ATPases, such as Na^+^/K^+^-ATPase, Ca^2+^-ATPase, and Mg^2+^-ATPase, which are essential for maintaining ionic gradients across the membrane [112,113]. In this method, cells are exposed in vitro to defined concentrations of nanoparticles and incubated with a reaction buffer containing ATP and the appropriate ionic cofactors (e.g., Mg^2+^, Na^+^, K^+^), and the enzymatic reaction is then terminated by the addition of trichloroacetic acid or another protein-denaturing agent. The amount of inorganic phosphate (Pi) released as a direct product of ATP hydrolysis is then determined colorimetrically, most commonly using ammonium molybdate or malachite green reagents. A reduction in ATPase activity, reflected by decreased Pi levels compared to untreated controls, is interpreted as an indicator of membrane damage or impaired ion transport caused by nanoparticle exposure. This method is sensitive, reproducible, and capable of detecting early sublethal cytotoxic effects, making it a valuable tool in the toxicological evaluation of nanomaterials for biomedical applications.

A summary of the in vitro techniques employed in MNPs toxicity assessment is presented in Table 3.

#### 3.1.7. Assessing the In Vitro Toxicity of Magnetic Iron Oxide Nanoparticles: Techniques and Experimental Findings

Colorimetric tetrazolium salt assays are widely employed in the assessment of MNPs toxicity, serving as the primary analytical approach for quantifying cell viability. These methods provide a rapid and sensitive measure of metabolic activity, which is commonly used as an indirect indicator of cellular health and survival. For example, Anuje et al. investigated the toxicity of PEG-coated Fe_3_O_4_ nanoparticles (10–20 nm) as a potential radiosensitizer in radiotherapy against L929 mouse fibroblasts and MCF-7 human breast cancer cells using the MTT assay [114]. Their results demonstrated that the tested MNPs were biocompatible toward L929 cells while inducing cytotoxic effects in MCF-7 cells. In another study, the MTT assay revealed that the toxicity of naked Fe_2_O_3_ nanoparticles (10 nm) toward HeLa cells was dependent on both their concentration and exposure time; however, in all cases, cell viability remained above 80% [115]. Similarly, Jarockyte et al. assessed the viability of NIH3T3 mouse embryonic fibroblasts using the XTT assay after exposure to naked Fe_3_O_4_ nanoparticles (15–20 nm) [116]. No cytotoxic effects were observed following 3 h and 24 h of incubation, with cell viability remaining at approximately 95%. The WST assay was also employed to evaluate and compare the toxicity of naked versus silica-functionalized MNPs in A549 and HeLa cells [117]. The results indicated that surface passivation of MNPs reduced their overall cytotoxicity.

In general, tetrazolium salt assays provide an initial insight into the potential toxicity of nanoparticles; however, cell survival following exposure to MNPs is also measured and confirmed using a variety of other in vitro techniques, including the LDH assay, trypan blue staining, Alamar Blue, and various inclusion dyes. For example, in the previously mentioned study employing the WST assay on A549 and HeLa cells, LDH leakage measurements conducted in parallel yielded results that closely correlated with the viability data, thereby supporting the reliability of the observed cytotoxicity trends [117]. In turn, Marcos-Campos et al. investigated the effects of various commercially available ferrofluids containing Fe_3_O_4_-core nanoparticles of different sizes (20–500 nm) on the survival of primary, monocyte-derived dendritic cells (DCs) [118]. In their work, trypan blue and PI staining consistently demonstrated a decreasing trend in DC viability from day 1 to day 5, with viability remaining above 65–70% on the fifth day of culture, thereby confirming that such complementary techniques can also be effectively applied to assess the cytotoxicity of magnetic nanoparticles. In another study, the toxicity of two types of superparamagnetic nanoparticles, silica-coated and oleic acid-coated, was evaluated against human neuroblastoma SH-SY5Y and human glioblastoma A172 cell lines using MTT, NRU, and Alamar Blue assays [119]. The authors emphasized, however, that caution should be exercised when interpreting results from colorimetric viability assays due to potential nanoparticle–assay interferences. This concern applies to all types of nanoparticles, not only MNPs. Indeed, one of the challenges in nanotoxicology is the potential interference of nanoparticles with classical in vitro cytotoxicity assays, which can lead to under- or overestimation of cell viability. Such effects may arise from optical disturbances, chemical interactions with assay reagents, or dye adsorption onto the nanoparticle surface. To minimize these effects, various strategies have been employed, including the use of control samples containing the same concentrations of nanoparticles but without assay reagents as blanks [120] and plate centrifugation prior to absorbance measurements [121].

The cytotoxicity of iron oxide nanoparticles is largely attributed to their ability to induce oxidative stress, which, in the case of MNPs, is frequently evaluated using the DCF assay as an indicator of reactive oxygen species generation, in combination with the assessment of GPx, SOD, and catalase activity, as well as the determination of NO levels [117,122,123,124,125,126]. Optical methods are currently the most commonly used approach for intracellular detection of ROS. However, they have several drawbacks, including the use of labels that may influence ROS formation, the multistep and complex nature of the techniques employed, and the inability to measure ROS within a single cell. Furthermore, most optical methods are unsuitable for long-term measurements due to the rapid inactivation of fluorescent dyes and their specificity toward only certain ROS species (mainly H_2_O_2_ [127]). An interesting solution in the case of magnetic nanoparticles has been proposed by Erofeev et al., who developed a rapid (<30 min) in vitro method for assessing MNP toxicity based on the measurement of intracellular ROS using a novel platinized carbon nanoelectrode with a cavity at the tip, integrated into a micromanipulator on an upright microscope [128]. Their results demonstrated significant differences in intracellular ROS levels in HEK293 and LNCaP cancer cells before and after exposure to 10 nm iron oxide MNPs. These findings contrasted with those obtained using conventional methods at the same MNPs concentration, where no differences in ROS levels were detected.

The comet assay is a widely applied method for assessing the genotoxic potential of magnetic nanoparticles. For example, Seo et al. applied this method to assess DNA damage in human hepatoma (HepG2) cells and demonstrated that a concentration of 5 μg/mL of DEX-coated MNPs caused significant DNA strand breaks [126]. In turn, Malvindi et al. demonstrated that bare Fe_3_O_4_ nanoparticles strongly increased DNA damage levels in A549 and HeLa cells, as evidenced by both tail length and the percentage of DNA in the tail, whereas silica-coated MNPs showed DNA damage values comparable to the control [117]. In other studies, comet assay results revealed time- and concentration-dependent increases in DNA damage induced by <100 nm Fe_2_O_3_ nanoparticles in the root meristem cells of *Allium cepa* after 24 and 96 h of exposure [51]. To assess the genotoxicity of MNPs, 8-OHdG can also used as a biomarker of oxidative DNA damage. For example, Cellai et al. investigated the generation of 8-dG (8-deoxyguanosine) in human hepatocarcinoma HepG2 cells. They observed no effects at any MNP concentration with incubation times ≤ 120 min, whereas adduct production increased significantly—up to six-fold—after ≥24 h of incubation compared to untreated cells, with the highest levels of 8-OHdG detected following treatment with 60–90 μg/mL of Fe_3_O_4_ nanoparticles for ≥24 h [129].

The above findings indicate that established in vitro methodologies provide an effective basis for assessing the toxicity of iron oxide nanoparticles; however, potential nanoparticle–assay interferences and model-specific limitations highlight the need to complement these approaches with additional, context-appropriate methods.

### 3.2. Ex Vivo and In Vivo Techniques

Compared to in vitro assays, in vivo studies are more time-consuming, expensive, and raise ethical concerns. Nevertheless, they offer the advantage of evaluating the overall toxicity of substances in living organisms, considering complex biological factors such as interactions with blood components (e.g., protein corona formation), biodistribution, and clearance [45]. Additionally, physiological ex vivo models using isolated tissues and organs enable the investigation of MNP-induced alterations at the tissue or organ level.

The initial step in toxicity evaluation typically involves behavioral observation of the test animals—commonly rats or mice—and monitoring of their body weight [87]. Ex vivo studies often include the measurement of serum biochemical markers indicative of liver and kidney function. These markers include alanine aminotransferase, aspartate aminotransferase, total bilirubin, direct bilirubin, total protein, albumin, globulin, albumin/globulin ratio, gamma-glutamyl transaminase, alkaline phosphatase, creatinine (CRE), blood urea nitrogen, and uric acid.

Hematological analysis is another widely employed approach in toxicity studies, encompassing the evaluation of red and white blood cells (RBCs and WBCs), as well as specific immune cell populations such as T lymphocytes and macrophages. Common assessments include the analysis of RBC and WBC morphology following nanoparticle exposure, along with the determination of the erythrocyte sedimentation rate (ESR) [85,87]. The ESR measures the rate at which erythrocytes settle at the bottom of a test tube under standardized conditions. An elevated ESR is a non-specific marker of inflammation and may also indicate infections, autoimmune disorders, or malignancies. Owing to its simplicity and clinical relevance, ESR is frequently used as a general screening parameter and is typically measured using automated analyzers.

These approaches are commonly employed in the evaluation of MNPs toxicity. For example, Woo et al. conducted ex vivo toxicity testing of three types of magnetic nanoparticles differing in surface charge (negative, positive, and neutral) in male Balb/c mice [130]. Blood was collected from the caudal vena cava, and serum was obtained by centrifugation. Biochemical markers were then measured to assess potential liver and kidney function alterations. In another study, the physiological and biochemical properties of erythrocytes were evaluated following single and repeated intravenous administrations of polyethylene glycol-coated superparamagnetic iron oxide nanoparticles (30 nm) in normotensive Wistar–Kyoto (WKY) and spontaneously hypertensive (SHR) rats [131]. Recently, Ruiz-Baltazar et al. performed a hemocompatibility assay using human red blood cells to evaluate the toxicity of Fe_3_O_4_–Ag decorated nanoparticles [132].

Histological and histopathological analyses are routinely conducted to examine tissue morphology in animals that are sacrificed following exposure to test substances. These methods facilitate the identification of structural changes in organs using light microscopy. Standard histological preparation involves tissue fixation, paraffin embedding, sectioning with a microtome to obtain thin slices, and subsequent staining—typically with hematoxylin and eosin (H&E). Hematoxylin stains nuclei blue, whereas eosin stains cytoplasmic and extracellular components pink, enhancing contrast and enabling the visualization of pathological alterations at both the cellular and tissue levels. Histopathological evaluation remains a cornerstone in MNPs toxicity studies due to the well-documented association between nanoparticle exposure and tissue damage [85]. For example, one study reported histopathological findings in organs collected from female Wistar rats following exposure to DMSA (dimercaptosuccinic acid)-coated magnetic nanoparticles (17 nm), demonstrating that all animals exhibited normal liver, spleen, cardiac tissue, and aortic artery histology 10 months after DMSA-MNPs administration [133]. In another study, mice intravenously exposed to ultra-small Fe_3_O_4_ nanoparticles (2.3–4.2 nm) underwent H&E staining of major organs, which revealed focal necrosis and acute inflammation in the heart and lungs, findings that correlated with elevated •OH levels and pronounced size-dependent acute toxicity [49]. Furthermore, Mîndrilă et al. investigated the effects of long-term intraperitoneal administration of salicylic acid-coated Fe_3_O_4_ nanoparticles in C57BL/6 mice. Detailed histopathological examination of the liver, complemented by Perls’ staining, revealed subcapsular changes and, at higher cumulative doses, perisinusoidal necrosis [134]. In addition to histopathological evaluation, biodistribution analysis can provide valuable insights into the potential toxicity of magnetic nanoparticles. For instance, ferromagnetic resonance (FMR) has been employed to detect and quantify DMSA-coated MNPs in the liver, spleen, kidney, and lungs, enabling precise assessment of nanoparticle accumulation in specific organs [133].

## 4. New Approach Methods (NAMs)

New Approach Methods, according to the U.S. Food and Drug Administration and European Medicines Agency, refer to a broad set of innovative and non-animal testing strategies developed to improve and partially or fully replace traditional in vivo toxicity assessment methods [135,136]. These include in vitro assays, in silico models (i.e., computational models or computer simulations), organ-on-chip technologies, and 3D bioprinted organ constructs. NAMs aim to enhance predictive power, reproducibility, and ethical acceptability of safety testing, especially in fields such as nanotoxicology, where traditional animal models may fail to replicate human-specific responses to nanoscale materials.

### 4.1. Advanced 3D Models in Nanotoxicology: Organoids and Bioprinting Approaches

Among the most promising NAMs are three-dimensional (3D) bioprinted organs and organoids, which serve as advanced ex vivo and in vitro models for studying the interaction between engineered nanomaterials and human tissues [137,138]. These systems are built by layer-by-layer deposition of biomaterials and living cells, recreating structural and functional characteristics of native tissues such as the liver, kidney, and heart.

#### 4.1.1. Three-Dimensional Bioprinting

3D bioprinting is an additive manufacturing technique that enables the precise spatial arrangement of cells, bioactive compounds, and signaling molecules to construct functional three-dimensional tissues and organs [139]. This technology allows for accurate architectural design, vascularization, and the co-culture of multiple cell types, making it particularly suitable for replicating complex tissue environments. 3D-bioprinted organs are typically fabricated on biocompatible scaffolds that mimic the extracellular matrix and support cellular organization, differentiation, and tissue-specific functionality. The constructs can be either acellular—guiding subsequent cellular colonization—or cell-laden, promoting tissue development from the outset. Prior to the advent of advanced biomanufacturing technologies, progress in developmental biology and cellular self-organization laid the groundwork for modern tissue engineering. While the formation of self-organizing multicellular units is a critical step, their integration into larger, functional tissue structures remains a major challenge. The synergistic combination of bioprinting and self-organization offers a promising strategy to enhance organoid formation, enabling high-throughput, anatomically relevant tissue fabrication using pluripotent or adult stem cells. Through geometric control enabled by bioprinting, stem cell aggregates can be directed to form more complex and physiologically accurate tissue models.

**Figure 3:**
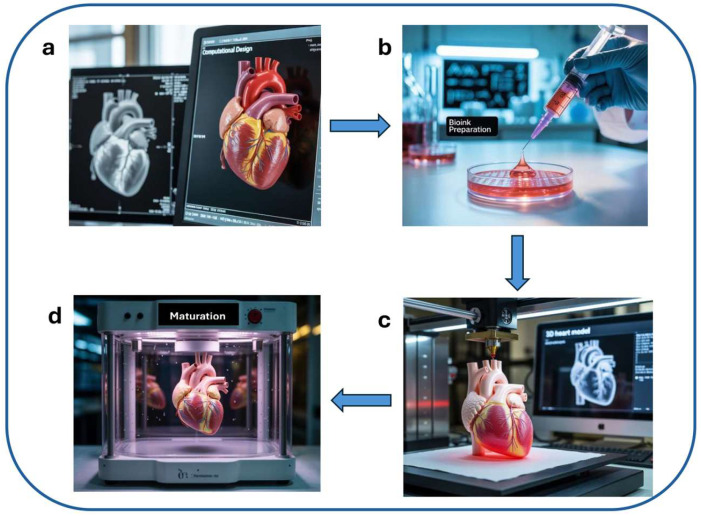
Conceptual diagram illustrating the main stages of 3D bioprinting of the organ: (**a**) computational design, (**b**) bioink preparation, (**c**) 3D printing of the organ, (**d**) functional maturation of the tissue. Created with Ideogram.ai. https://ideogram.ai/t/my-images (accessed on 29 July 2025).

The fundamental stages of 3D organ bioprinting are conceptually illustrated in Figure 3.

The use of perfusable, vascularized 3D constructs enables simulation of physiologically relevant conditions, enhancing the translational accuracy of experimental findings. As acknowledged by the OECD (Organization for Economic Co-operation and Development) and EURL ECVAM (European Union Reference Laboratory for alternatives to animal testing/European Centre for the Validation of Alternative Methods), these technologies are now recognized as part of the next-generation toolbox of regulatory-compliant NAMs, offering reliable alternatives to conventional animal-based toxicity testing.

Various bioprinting techniques have been developed to construct functional tissue-like structures with increasing spatial precision and biological relevance. Among them, four major strategies are currently employed, each with distinct advantages and limitations. Inkjet-based bioprinting is a contactless technique that uses thermal or piezoelectric nozzles to dispense droplets of bioink—typically composed of hydrogels mixed with cells—according to a predesigned pattern [140]. Laser-assisted bioprinting avoids physical nozzle contact by employing laser-induced forward transfer or laser direct-write techniques [141]. This enables high-resolution deposition of viscous biomaterials and dense cell suspensions with minimal shear stress. Extrusion-based bioprinting, the most widely used approach, involves the continuous extrusion of bioink under pneumatic or mechanical pressure through a nozzle [142]. It supports a broad range of viscosities and high cell densities, making it suitable for printing more robust and volumetric tissue structures. Photo-curing bioprinting, including stereolithography (SLA) and digital light processing (DLP), relies on light-induced polymerization to cure photosensitive bioinks in a layer-by-layer fashion [143]. This technique offers high printing resolution, avoids nozzle-related issues, and allows rapid production regardless of structural complexity. Its ability to guide cell alignment and self-organization makes it especially promising for organoid development. Each method offers specific benefits depending on the targeted tissue, desired resolution, and biological complexity.

The use of 3D bioprinted models in studies on magnetic nanomaterials remains relatively limited; however, recent reports highlight their potential for assessing the cytotoxicity and biocompatibility of magnetic nanoparticles. For example, Theus et al. employed GelMA (gelatin-methaacryloyl)-based 3D-bioprinted scaffolds containing various concentrations of superparamagnetic iron oxide nanoparticles (0–500 µg/mL) to compare toxicity outcomes in 2D versus 3D environments, showing that the 3D matrix attenuated some cytotoxic effects observed in conventional monolayer cultures [144]. Similarly, Ning et al. developed a perfusable 3D-bioprinted pulmonary vein model to investigate the effects of MNPs and MNPs–rapamycin conjugates on endothelial cell viability and function, using assays such as AlamarBlue, Live/Dead staining, and flow cytometry [145].

#### 4.1.2. Organoids

**Figure 4:**
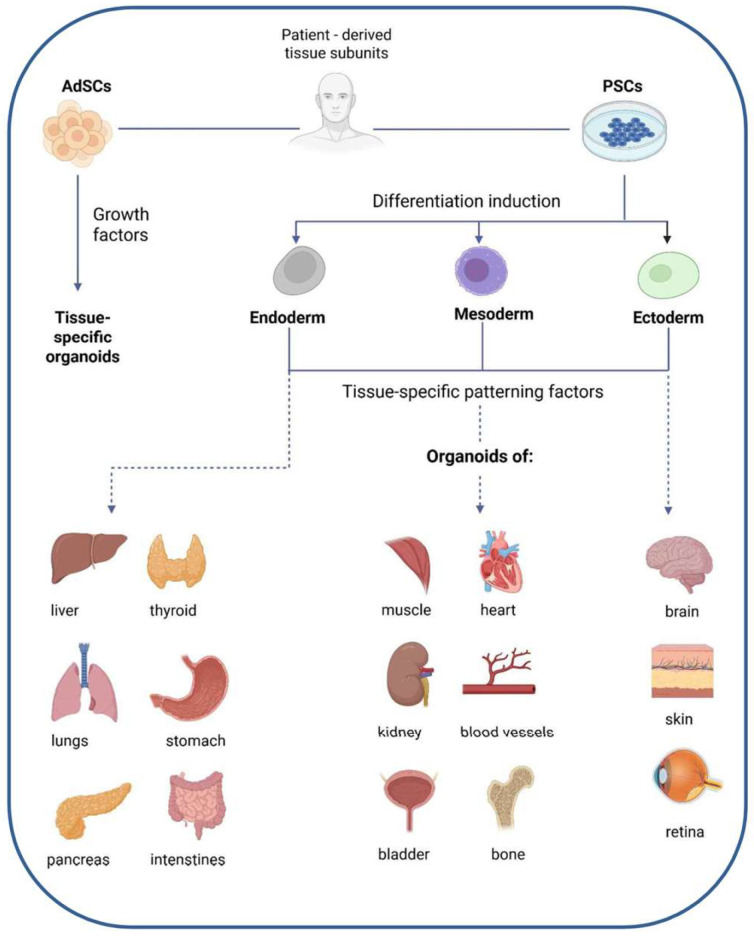
Organoid generation through guided self-organization and tissue-specific differentiation. Created in Biorender.com. Julia Nowak-Jary. (2025) https://BioRender.com/ (accessed on 29 July 2025).

Organoids are 3D in vitro culture systems that closely mimic their tissue or organ counterparts in vivo [146]. They are derived from either pluripotent stem cells (PSCs) or adult stem cells (AdSCs) and self-organize into miniature, organ-like structures. PSC-derived organoids differentiate through the germ layers (endoderm, mesoderm, or ectoderm) under the influence of specific growth signals to develop into organ-specific phenotypes (Figure 4). In contrast, AdSC-derived organoids originate from isolated tissue-specific stem cell populations embedded in an extracellular matrix (ECM) and are expanded using targeted growth factors. By replicating the cellular composition and functional characteristics of native human organs, organoids serve as valuable intermediate models bridging the gap between animal studies and clinical research. The use of organoids offers several advantages, including model individualization, relatively rapid generation, suitability for high-throughput screening of drugs or genetic modifications, toxicology and the potential for gene editing. However, current organoid models still face notable limitations, such as incomplete recreation of the in vivo microenvironment, limited vascularization, discrepancies in size and spatial organization compared to whole organs, and the lack of fully established co-culture systems with multiple cell types.

Parallel to bioprinted systems, organoid models are also applied in magnetic nanomaterial research, offering a more physiologically relevant representation of tissue architecture, cellular heterogeneity, and in vivo-like functions. For instance, Palzer et al. demonstrated that MNPs-containing magnetoliposomes exhibited no significant cytotoxicity in patient-derived pancreatic ductal adenocarcinoma organoids, with a marked decrease in viability observed only upon exposure to an alternating magnetic field [147]. In another study, Sun et al. evaluated MNPs-labeled pancreatic islet organoids for magnetic particle imaging and confirmed that nanoparticle incorporation did not impair cell viability or insulin secretion [148]. These findings underscore the promise of organoid platforms as physiologically relevant and versatile tools for evaluating the safety and biological interactions of magnetic nanoparticles.

While organoid and bioprinting models are now commonly used for evaluating the effects of various types of nanoparticles, their application to magnetic nanomaterials is still in its early stages. Nonetheless, there are no fundamental obstacles preventing the use of these techniques for assessing the toxicity of MNPs. Given that 3D bioprinting and organoids are emerging New Approach Methodologies, a steady increase in studies evaluating MNPs toxicity with these platforms is expected in the coming years.

### 4.2. In Ovo Models

**Figure 5:**
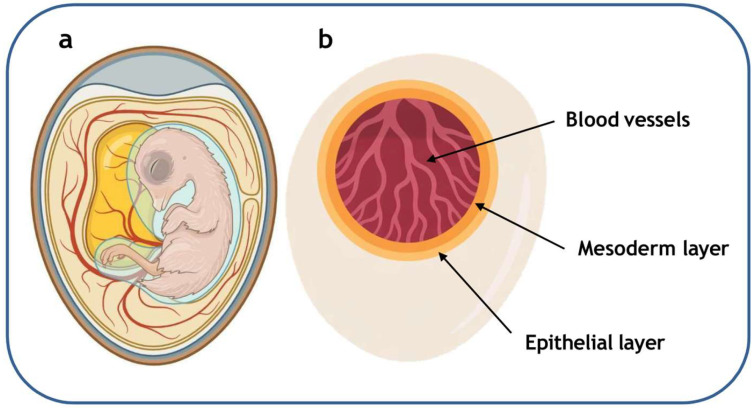
Graphical illustration of the chicken embryo (**a**) and the CAM (**b**). The CAM comprises two different layers and a blood vessel system. Created in Biorender. Julia Nowak-Jary. (2025) https://BioRender.com/ (accessed on 29 July 2025) and Ideogram.ai. https://ideogram.ai/t/my-images (accessed on 29 July 2025).

The in ovo method entails the administration of test substances into fertilized eggs containing live embryos at advanced developmental stages, most commonly around the 18th day of incubation (Figure 5a). Nanoparticles and other test agents can be delivered either directly into the embryo or into the egg’s air chamber. In embryo-based models, nanoparticle toxicity is typically evaluated using techniques such as the comet assay, hemolytic assay, chicken embryonic chorioallantoic membrane (CAM) assay, and measurements of oxidative stress markers [149] with the chicken embryo serving as the predominant experimental model in such studies [150].

The chorioallantoic membrane (CAM) of the chicken embryo (CE) is a crucial structure that supports embryonic development by facilitating efficient gas exchange, nutrient uptake, and the removal of metabolic waste products [151]. It arises from the fusion of two extraembryonic membranes: the chorion, which represents the outermost layer derived from the trophoblast and initially envelops the embryo, and the allantois, an outpouching of the embryonic hindgut that expands into the extraembryonic cavity and subsequently merges with the chorion to form the CAM.

In its mature stage, the CAM is organized into three distinct layers (Figure 5b). The outer surface is lined with an epithelial layer composed of epithelial cells. Beneath it lies the mesodermal layer, containing blood vessels and connective tissue, and deeper still, there is a dense vascular network that ensures the supply of oxygen and nutrients to the developing embryo, constituting a fundamental platform for angiogenesis studies [151].

CAM technique offers a potential alternative to traditional in vivo studies by reducing animal discomfort, experimental complexity, and associated costs [150]. In ovo models are cost-effective and straightforward, requiring only inexpensive egg acquisition and minimal maintenance [152,153]. The chorioallantoic membrane, an extra-embryonic and highly vascularized structure, provides convenient access for experimental manipulation. Chicken embryo development spans 21 days from the initiation of incubation to hatching. The CAM appears between embryonic days 4 and 5 and functions as a transparent, vascularized membrane in direct contact with the eggshell, supporting gas exchange as the embryo’s primary respiratory organ. Moreover, the CAM model often bypasses the need for formal ethical approval, as chick embryos are not classified as living animals before embryonic day 17 (EDD—embryonic development day) in most jurisdictions. Despite these advantages, the in ovo model has inherent limitations, including incomplete knowledge of interactions between diverse cell types and organs, as well as the inability to fully replicate long-term physiological effects observed in human exposure scenarios. Moreover, the CAM is sensitive to changes in pH, osmolarity, keratinization, and oxygen tension that may occur during shell opening or sealing procedures [154]. It is also crucial to conduct all experiments at the same EDD, as CAM development is characterized by rapid morphological changes [155].

Although in ovo studies most frequently employ the chicken embryo as a model organism, zebrafish embryos have also been utilized for evaluating the toxicity of MNPs. For instance, Jurewicz et al. utilized this second model to investigate the concentration—dependent toxicity of MNPs conjugated with the fluorescent dye Congo Red [82]. In turn, Patel et al. investigated the neurotoxic potential of Fe_3_O_4_ nanoparticles using the in ovo chicken embryo model. The study aimed to assess concentration—dependent embryotoxic and neurotoxic effects following exposure to varying doses of MNPs [156]. In a related experiment, MNPs in the form of maghemite (Fe_2_O_3_) and magnetite (Fe_3_O_4_) were administered into fertilized chicken eggs at doses ranging from 100 to 2500 ppm to evaluate their teratogenic and neurodevelopmental impact. The results demonstrated morphological brain alterations—including neuronal necrosis, edema, and vacuolation—as well as structural malformations such as failure of abdominal wall closure and cardiac displacement [157]. Furthermore, a separate study employed the CAM model to investigate both the biocompatibility and embolic potential of aqueous dispersions of Fe_3_O_4_ nanoparticles functionalized with salicylic acid, synthesized in various sizes [158]. The nanoparticles were intravenously administered into the CAM vasculature, and their biological behavior was evaluated with and without the application of an external magnetic field to assess the risk of vascular occlusion. This study not only emphasized the relevance of nanoparticle surface chemistry and size in determining vascular interactions but also demonstrated the suitability of the CAM model as a rapid and sensitive platform for preclinical toxicity screening and nanoparticle design optimization.

Despite the above examples, the current body of literature on the embryotoxicity of magnetic nanoparticles remains limited. Existing data provide valuable insights but are often confined to specific nanoparticle formulations, concentrations, or exposure windows, thereby constraining broader conclusions. Consequently, comprehensive and systematic investigations are required to assess long-term developmental effects, dose–response relationships, and underlying mechanisms of toxicity across different nanoparticle types.

### 4.3. Imaging-Based Cytometry: Visualizing Nanoparticle—Cell Interactions

Flow cytometry is a widely adopted and highly quantitative technique commonly used in nanotoxicology to assess nanoparticle-induced effects on cells in suspension, such as blood or fluid-derived systems [159,160]. Its ability to rapidly analyze thousands of individual cells per second, while simultaneously detecting multiple fluorescence markers, and to isolate specific subpopulations via high-purity cell sorting makes it a powerful tool in evaluating nanoparticle cytotoxicity, oxidative stress, and immunological responses. The technique supports multiparametric detection of markers associated with apoptosis, reactive oxygen species, cell cycle arrest, and immune activation, allowing for comprehensive profiling of nanoparticle effects. The method is particularly effective in studies where nanoparticle exposure requires high-throughput, multiparametric data from single-cell suspensions.

For adherent cell models or tissue-based systems that are often used to mimic in vivo nanoparticle interactions, scanning image cytometry is more suitable. This approach allows quantitative imaging of fixed or live cells directly on a substrate, preserving spatial architecture and cell morphology—features critical for assessing how nanoparticles interact with cell membranes, cytoskeletons, or multicellular structures [160,161,162]. These systems often integrate automated image analysis software, enabling objective quantification of fluorescence intensity, cell shape alterations, and subcellular localization of nanoparticles. Although slower than flow cytometry, image cytometry provides essential insights into cell behavior and toxicity at the structural level.

While most image cytometry systems operate in 2D, recent advancements in nanotoxicology emphasize the importance of analyzing 3D cellular models, which better reflect human physiological conditions [160]. Acquiring reliable 3D imaging data, however, remains technically demanding due to challenges such as focal plane alignment and photobleaching during fluorescence detection. Advanced imaging techniques, including confocal laser scanning microscopy or structured illumination microscopy (SIM) are increasingly used to overcome these limitations and improve spatial resolution in 3D samples. For fixed 3D cultures, the use of anti-fade reagents improves signal stability, enabling more accurate characterization of nanoparticle distribution and toxic effects within tissue-like structures.

Image cytometry has emerged as a powerful tool enabling quantitative, high-content analysis of cellular responses to nanoparticles. Eustaquio and Leary conducted a comparative analysis of flow cytometry and scanning image cytometry to assess the nanotoxicity of MNPs [163]. Both techniques enabled the quantification of apoptosis, necrosis, and changes in cell morphology, but scanning image cytometry proved particularly advantageous for analyzing adherent cells, as it preserved spatial architecture and allowed for more precise detection of localized fluorescence signals. This approach highlighted the complementary strengths of image-based methods in mechanistic studies of nanoparticle–cell interactions. In subsequent work, the same researchers applied scanning image cytometry to investigate the cellular response to nanobarcoded MNPs functionalized with oligonucleotides [164]. Their methodology enabled simultaneous single-cell measurements of nanoparticle uptake, viability, apoptosis, necrosis, and ROS generation. By integrating high-resolution imaging with quantitative fluorescence analysis, the study demonstrated how image cytometry can offer deep insights into nanoparticle-induced effects while maintaining cellular morphology and spatial distribution. Friedrich et al. demonstrated that flow cytometry can be effectively used to estimate intracellular uptake of MNPs by mammalian cells, offering a rapid and reliable analysis method [165]. Polasky et al. further employed this technique using FITC (fluorescein isothiocyanate)-labeled DEX-coated MNPs in THP-1 monocytes and monocyte-derived macrophages, assessing nanoparticle uptake via fluorescence and evaluating monocyte activation through surface markers CD14, CD11b, and CD86 (clusters of differentiation 14, 11b, and 86, respectively) [166].

From a methodological standpoint, both flow cytometry and image-based cytometry are primarily classified as in vitro techniques, as they rely on the analysis of cultured cells outside the organism. However, flow cytometry may occasionally be applied to freshly isolated ex vivo samples. Importantly, their high-throughput capacity and ability to reveal underlying biological mechanisms position them within New Approach Methodologies, which aim to reduce or replace animal testing through advanced in vitro and computational strategies.

## 5. Discussion and Future Perspectives

MNPs represent a rapidly expanding area of research with substantial potential in drug delivery and theranostics. The need for precise therapeutic targeting, together with the growing challenge of drug resistance in certain cancers, underscores the rationale for nanoparticle-based strategies. Importantly, MNPs can differentially affect healthy and malignant cells, enabling selective elimination of tumor cells—an especially desirable feature in oncology. Moreover, magnetic nanoparticles can be directed to specific anatomical sites using an external magnetic field, thereby improving targeting precision and minimizing off-target effects. In addition, hyperthermia induced by an alternating magnetic field can be combined synergistically with chemotherapy to enhance overall efficacy. Nevertheless, despite these advantages, comprehensive evaluations of potential toxicity remain essential, covering both acute and chronic effects to ensure that the therapeutic benefits are not outweighed by risks to patient safety or unintended biological consequences.

Nanotoxicity is multifactorial, reflecting contributions from particle size, shape, surface chemistry, surface charge, protein corona formation, and in vivo biodistribution and clearance. Technical parameters—dose, exposure duration and frequency, and even synthetic precursors—also modulate apparent toxicity. Notably, nanoparticles with similar physicochemical characteristics may display different toxicological profiles across experimental models, emphasizing the importance of context. Apparent contradictions in the literature frequently stem from batch-to-batch variability (e.g., coating density/grafting heterogeneity, partial oxidation of Fe_3_O_4_ to γ-Fe_2_O_3_ during storage), as well as from differences in hydrodynamic size, aggregation/sedimentation, and corona composition measured in the actual exposure medium rather than in buffer alone. Disparities in dosimetry (administered vs. delivered dose), unit systems (µg Fe/mL vs. mM Fe), and plate geometry or mixing can likewise invert “toxicity rankings,” particularly for adherent cells sensitive to sedimentation artifacts. Model selection adds further divergence: cell lineage, passage number, differentiation state, and baseline antioxidant capacity (and, in vivo, species, route, and kinetics) strongly shape outcomes, as do exposure timepoints and oxygen tension. Methodological factors—assay interference by particles (optical quenching/absorbance), unrecognized endotoxin contamination, or non-orthogonal endpoints—can also yield conflicting readouts. Mitigation requires rigorous physicochemical characterization in exposure media (Dh/PDI/ζ), harmonized reporting and dose metrics, control of endotoxin and NP–assay interference, within-class comparisons under matched conditions, and the use of orthogonal biological endpoints to validate conclusions.

Mechanistic convergence across studies suggests that MNPs toxicity is driven primarily by oxidative stress and downstream injury to cellular organelles. Acidic endosomal/lysosomal processing can accelerate iron dissolution from cores (or incompletely passivated surfaces), increasing labile iron and catalyzing ROS generation; transcriptomic and serum-biochemistry datasets consistently point to oxidative stress pathways under relevant conditions [47,48,56,57,58]. Mitochondria are frequent targets: size-defined Fe_3_O_4_ nanoparticles have been shown to trigger mitochondrial ROS, bioenergetic collapse, and necrotic outcomes in specific cell lines, while larger particles may disrupt plasma-membrane integrity (e.g., LDH release) [52]. Depending on iron handling and antioxidant reserves, these events can be coupled to lipid peroxidation and ferroptotic signaling, with lysosomal damage and ferritinophagy further modulating intracellular iron pools [167,168,169,170]. Collectively, these mechanisms align with model-dependent differences observed in vivo (e.g., zebrafish gill vs. liver responses) and in vitro (e.g., charge- and coating-dependent genotoxicity), reinforcing the need to interpret results within a single MNP class under matched media and dose metrics [61,62,63]. This synthesis highlights oxidative stress and organelle dysfunction as unifying mechanisms of MNP toxicity, providing a mechanistic framework for interpreting disparate findings across models.

When developing MNPs for biomedical use, design principles commonly aim to minimize nanoparticle-induced toxicity. A widely adopted approach is surface functionalization with biocompatible polymers such as PEG), which can reduce toxicity by decreasing nonspecific interactions with cell membranes and limiting uptake. Appropriate coatings may also suppress acid-promoted iron release, thereby mitigating oxidative stress. Morphology is another critical design parameter: shape and surface curvature govern interfacial interactions and may influence dissolution behavior. Although smaller radii of curvature are generally less stable and more prone to dissolution, drawing universal toxicity rankings based on shape (e.g., rods < spheres) is problematic without matched media and dosimetry; morphology–toxicity relationships remain formulation- and model-dependent.

One strategy to offset oxidative stress involves co-administration of antioxidants during MNP-based therapy. Ucar et al. reported that ulexite (18.75 mg/L) attenuated oxidative stress in brain tissue [171], while quercetin-conjugated MNPs promoted neurogenesis without detectable toxicity [172]. Proposed mechanisms include metal chelation, modulation of iron-homeostasis genes, radical scavenging, and partial suppression of Fenton and Haber–Weiss reactions.

Despite these general principles, each engineered nanoparticle requires an independent, purpose-specific toxicity assessment. In vitro studies remain indispensable for screening and dose-setting, but in vivo evaluation is necessary to capture whole-organism pharmacokinetics, immune interactions, and long-term outcomes within complex physiological environments.

In parallel with advances in particle design, predictive and ethically aligned testing frameworks—NAMs—are increasingly being adopted. These include in vitro, in silico, ex vivo, and in ovo systems that reduce reliance on animal testing while providing mechanistic, higher-throughput data. Among these, the chorioallantoic membrane model offers a cost-effective, vascularized, and immunologically immature system for early-stage assessment of angiogenesis, inflammation, and tissue integration [153]. Three-dimensional (3D) bioprinting and organoid platforms enable spatially organized, multicellular microenvironments that better approximate human tissue physiology [140,143]. Human liver or neural organoids combined with MNPs can recapitulate uptake, accumulation, and aspects of toxicity not captured by monolayers [173]. Although outstanding challenges remain in long-term exposure, systemic biodistribution, and regulatory acceptance, NAM-based models—especially CAM and bioprinted constructs—when combined with computational simulations and omics-scale profiling provide a forward-looking path toward safer nanomedicines aligned with human relevance, scalability, and reproducibility [174,175].

At the same time, NAMs carry important limitations that must be acknowledged to avoid over-interpretation. Organoids and 3D-bioprinted tissues often lack full vascular perfusion, mature immune components, and long-term homeostatic turnover; batch-to-batch variability, matrix composition, and maturation state can shift nanoparticle transport, corona formation, and injury thresholds [140,143,173]. CAM assays provide rapid vascular readouts but have a constrained developmental window, interspecies differences, and limited recapitulation of mammalian pharmacokinetics [153]. Imaging flow cytometry (IFC) offers single-cell, high-content morphology and localization data, yet it may be confounded by nanoparticle autofluorescence, quenching, spectral overlap/compensation, and scattering artifacts without rigorous controls and appropriate reference materials [162,176]. Finally, cross-platform dosimetry (µg Fe/mL vs. mM Fe; delivered vs. administered dose), medium-dependent aggregation/sedimentation, and non-standard endpoints hinder aggregation of evidence across laboratories [174,175]. Mitigation strategies include harmonized SOPs (standard operating procedures), inter-laboratory ring studies, use of well-characterized reference nanoparticles, reporting of hydrodynamic size and polydispersity in exposure media, orthogonal endpoints (viability, membrane integrity, genotoxicity), and explicit uncertainty analyses [174,175,177,178].

Taken together, MNPs-enabled precision therapies remain promising, particularly where magnetic guidance and hyperthermia can be rationally integrated with pharmacology. Yet progress depends on closing mechanism-to-outcome knowledge gaps (iron handling, ROS/mitochondria/lysosomes/ferroptosis), strengthening quantitative dosimetry, and deploying NAMs alongside targeted in vivo studies under transparent, standardized reporting. This balanced approach is essential for translating laboratory promise into clinically robust and safe nanomedicines.

## Figures and Tables

**Table 1:** Summary of MNP toxicity by size and shape.

Core	Shape	Core Size (nm)	Coating	Model	Key Outcomes	Ref
Fe_3_O_4_	Spherical	10, 20, 30, 40	Amphiphilic polymers with carboxylic acid	Female KuMing mice	No significant change in 7 hepatic markers across sizes; 10 nm MNPs increased TBIL/DBIL, and lowered ALP; smaller MNPs persisted longer in blood; WBC/PMN changes; size-dependent hepatic gene expression (oxidative stress, immune, iron transport, metabolism, apoptosis) without overt toxicity	[47]
Fe_3_O_4_	Spherical	10, 20, 40	AEAPS ^1^	Male Sprague Dawley rats	Distinct biochemical responses by size; LDH (partial negative) and urea (partial positive) correlations with size	[48]
Fe_3_O_4_	Spherical	40, 80	BSA ^2^; PEG ^3^ derivatives	Human fibroblasts and U251 glioblastoma cells	Toxicity driven by surface chemistry, dose, exposure time; variable diameters of MNPs did not cause significant changes (except proliferation assay)	[44]
Fe_3_O_4_	Spherical	2.3, 4.2, 9.3	No coating molecules	Male ICR mice	2.3/4.2 nm highly toxic/lethal; 9.3 nm no apparent toxicity; mechanism attributed to oxidative stress	[49]
Fe_3_O_4_	Spherical	6, 9, 14	No coating molecules	Human hepatoma (SH-Hep-1, Hep3B) cells	6 nm least cytotoxic; 9 nm caused mitochondrial ROS and necrosis; 14 nm caused membrane damage (LDH release)	[50]
γ-Fe_2_O_3_	Spherical	<50, <100 (hydrodynamic diameter)	No coating molecules	Transheterozygous larvae of Drosophila melanogaster	MNPs < 100 nm: no genotoxicity; <50 nm: genotoxicity at 1 and 10 mM	[51]
γ-Fe_2_O_3_	Rod-shaped vs. spherical	Nanorodes: 50–100 (diameter), >500 (length) Spheres: <5000 and <50	No coating molecules	Murine macrophage cel line RAW 264.7	Nanorods: higher uptake than spheres; higher accumulation led to necrosis in non-tumorigenic cells	[52]
Not specified	Nanocubes	40	DSPE ^4^-PEG	Mouse monocyte macrophage RAW264.7 cell line	Low cytotoxicity up to 0.5 mg Fe/mL	[53]
Fe_3_O_4_	Nanoworms	Nanoworms: 65 (length) Spheres: 6	HA ^5^ (hyaluronian)	CD44 expressing mouse macrophage RAW264.7 cells	Nanoworms elicited lower inflammatory response than spherical nanoparticles	[54]
Fe_3_O_4_	Spheres, nanoworms, nanorodes, magnetic beads	2.8–21.6	PVA ^6^ Polyvinyl alcohol	L929 fibroblasts	Abnormal morphology (cell shrinkage); toxicity order: beads < nanoworms < nanospheres	[55]

^1^ AEAPS—*N*-(2-aminoethyl)-3-aminopropyl trimethoxysilane; ^2^ BSA—bovine serum albumin; ^3^ PEG—polyethylene glycol; ^4^ DSPE—1,2-distearoyl-sn-glycero-3-phosphoethanolamine; ^5^ HA—hyaluronian; ^6^ PVA—polyvinyl alcohol.

**Table 2:** Determinants of apparent MNPs toxicity organized by MNPs-based, cell-based, and technical factors.

Category	Factor	Why It Matters	Representative Examples and Models	Typical Direction of Effect	Controls/Standardization
MNPs	Core size	Alters surface-to-volume ratio, dissolution kinetics, biodistribution, and intracellular processing.	BSA ^1^-coated Fe_3_O_4_ 40 vs. 80 nm largely driven by surface chemistry/dose/time rather than diameter (human fibroblasts and U251 glioblastosoma cells) [44]; PEG ^2^-coated 10–40 nm spheres in female KuMing mice showed no size-dependent shifts in seven standard hepatic markers versus controls, but 10 nm particles increased TBIL/DBIL and lowered ALP; smaller particles also persisted longer in blood and modulated WBC/PMN without overt toxicity [47]; 10/20/40 nm silica-coated MNPs in male Sprague Dawley rats (LDH/urea correlations)—the smallest particles showed the highest toxicity [48]; ultrasmall Fe_3_O_4_ (2.3–4.2 nm) were lethal in male ICR mice vs. 9.3 nm without acute toxicity [49]; Fe_3_O_4_ 6/9/14 nm showed model-specific mechanisms in human hepatoma SK-Hep-1/Hep3B (mitochondrial ROS vs. membrane damage) [50].	Smaller cores can increase reactivity and toxicity in vivo; in vitro relationships may invert across endpoints and lines.	Report core size (TEM/XRD), Dh ^15^ and PDI ^16^; compare within a single core chemistry under matched media and exposure.
	Particle shape/aspect ratio	Modulates membrane contact area, uptake pathways, and intracellular fate.	Nanorods showed higher uptake and accumulation than spheres in mouse monocyte–macrophage RAW 264.7 line [52]; DSPE ^3^-PEG-coated nanocubes showed low cytotoxicity (≤0.5 mg Fe/mL) in mouse monocyte–macrophage RAW 264.7 line [53]; HA ^4^-nanoworms (mouse monocyte–macrophage RAW 264.7 line) elicited lower inflammatory response than spherical HA-MNPs (CD44) [54]; L929 fibroblasts: MNPs (with the same molarity) presented toxicity order: nanospheres.beads < nanoworms < nanospheres [55].	Higher aspect ratio often increases uptake and injury; effects remain formulation- and model-dependent.	Compare only within the same core/coating; quantify aspect ratio; keep iron dose constant across shapes.
	Surface coating (chemistry)	Governs iron release in acidic compartments, protein corona, membrane interactions, biodistribution.	Acidic dissolution hierarchy: PLGA ^5^ slower (~9.56%) vs. bare (~15.3%) vs. DEX ^6^ (20.4%) and PVA ^7^ (21.9%) over 72 h in endosomal-like media [57]; bare MNPs exerted greater toxicity than starch-coated MNPs in adult Zebrafish gill; in contrast, starch-MNPs triggered more severe damage on liver [62]; PVA and poly(arabic acid) coatings did not penetrate/destabilize DPPC bilayer [64]; PVA coating-MNPs reduced antigen processing/CD4^+^ T-cell stimulation in dendric cells (DCs) [66]; DEX/PEG ^6^ were less cytotoxic to aortic endothelium than uncoated (lower ROS formation) [81].	Coatings can attenuate or exacerbate apparent toxicity depending on dissolution kinetics and interfacial behavior.	Specify polymer identity/MW ^17^ and grafting density; characterize corona; interpret within one coating class.
	Surface charge (ζ-potential)	Electrostatics control adhesion to negatively charged membranes, uptake, and intracellular trafficking.	Amine (APTMS ^8^) and TEOS ^9^/APTMS-coated MNPs (+) increased membrane attachment and induced dose-dependent DNA damage in fibroblasts and fibrosarcoma normal cells; bare/TEOS-coated MNPs (−) did not in the same assays [65]; amine-Si-coated MNPs (+) were more cytotoxic than neutral azide-Si-coated, while carboxyl-Si-coated MNPs (−) were minimally cytotoxic (rat alveolar macrophage and human colonic adenocarcinoma cells) [67].	Positive charge often increases cytotoxicity and genotoxicity; neutral/negative usually milder (toxicity system- and exposure-dependent)	Report ζ-potential in the exposure medium; maintain ionic strength/serum constant; compare within a given charge class.
	Colloidal behavior (Dh, aggregation, sedimentation)	Delivered dose and cell contact depend on Dh/agglomeration; can invert apparent toxicity rankings.	PLGA-PEG-coated MNPs caused sedimentation effect—after 24 h viability loss in human lung adenocarcinoma epithelial cell line A549 due to slow sedimentation/film over cells [60]; general note that aggregation depends on charge/coating/medium composition [59].	Large/agglomerated Dh can increase apparent toxicity via sedimentation artifacts rather than intrinsic chemistry.	Measure Dh/PDI in relevant media; pre-disperse consistently; control plate geometry and mixing; normalize to delivered dose.
Experimental models	Cell lineage/phenotype	Different uptake, antioxidant capacity, and membrane properties drive model-specific responses.	Human lung adenocarcinoma epithelial A549 epithelial cells: PLGA-PEG-coated MNPs—sedimentation effect [60]; human fetal osteoblast (hFOB) and human breast adenocarcinoma (MCF-7) cell line: bare most cytotoxic; HEC ^10^/PVP ^11^-coated mild toxicity; partial NCC ^12^ coverage increased toxicity [61]; human umbicilal artery smooth muscle cells (HUASMCs): ~10 nm cores with DEX/PVA/PLGA showed >70% viability without morphology changes [57]; DCs showed reduced antigen processing with PVA-MNPs [66]; porcine aortic endothelial cells (PAEC) less sensitive to DEX/PEG-coated vs. uncoated [81].	Phagocytic/immune cells often show stronger responses; endothelial/mesenchymal responses vary by coating.	Use panels spanning epithelial, endothelial, immune, and stromal lines; report passage, origin, and culture conditions.
	Organ/tissue context and species/stage	Organ physiology and developmental stage alter exposure routes, clearance, and pathway activation.	Adult zebrafish: bare MNPs more potent in gill; starch-coated more hepatic; overlapping pathways by RNA-seq [62]; zebrafish embryos: CR ^13^-MNPs non-teratogenic at tested levels; higher toxic effect on zebrafish larvae [82].	Organ- and stage-specific effects; embryo assays may reveal developmental hazards not seen in adults.	State species, stage, organ; align exposure metrics; avoid cross-species generalization without caveats.
Technical factors	Dose metrics and exposure duration	Apparent toxicity scales with concentration/time but nonlinearly across endpoints and models.	All formulations produced a concentration- and time-dependent decrease in viability of human lung adenocarcinoma epithelial A549 cells [60]; concentration- and time-dependent toxicity of core/shell GoldMag nanoparticles in human umbilical vein endothelial cells (HUVEs) [69]; concentration- and time-dependent toxicity of PVP-MNPs in human coronary artery endothelial cells (HCAEs) [70]; zebrafish embryo assays: minimal toxic effect at 200 μg/mL of CR-MNPs; toxic effects at high concentration (800 μg/mL) [82].	Longer exposure and higher dose usually increase toxic effects; kinetics and accumulation modulate outcomes.	Report iron mass (µg Fe/mL), molarity (mM Fe), and surface area where possible; include multiple time points.
	Medium composition/assay system	Ions, proteins, and buffer components shape aggregation, corona, and membrane interaction; assay readouts vary by platform.	Aggregation/agglomeration depends on i.a. medium composition and controls delivered dose [59]; bare (44 nm) and PEG-coated MNPs (76 nm) were ≥2× less cytotoxic than PEG-PLGA-MNPs under matched conditions [60]; DPPC bilayer studies showed that PVA/PAA ^14^-coated MNPs are non-disruptive at early contact [63]; genotoxicity outcomes differed across assays (Ames vs. mammalian panels) [62].	Serum and ionic strength can mask or enhance effects; different assays capture different hazard facets.	Fix serum %, ionic strength, and pH; run orthogonal assays (e.g., viability + LDH + genotoxicity); document media and buffers.
	Rout of administration	Pulmonary, oral, intravenous injection	Pulmonary administration of MNPs in Wistar rats induce lung inflammations [72]; Oral: no observable changes in the digestive system [74]; possible hormonal imbalances [78]; intravenous injection of MNPs associated with adverse effects: oxidative stress and DNA damage in the heart [79]; necrosis of cardiac muscle tissue [80]; hemolysis and elevated AST and ALT levels [83]; apoptosis in human skin fibroblasts [84].	Pulmonary: acute lung inflammation. increase in BALF LDH/total protein; oxidative stress in lung; histologic inflammatory changes; Oral: generally low overt GI pathology; Intravenous injection: Rapid RES sequestration (liver/spleen/lymph nodes); potential hemolysis/coagulation activation; transient hemodynamic effects; increase in liver enzymes; cardiac oxidative stress/DNA damage for certain coatings/doses	Inhalation: OECD TG 412/413, NP characterization, sham/positive controls. Instillation: standardized dose/dispersion, endotoxin-free (LAL). Oral: OECD TG 420/407/408, vehicle control, GI monitoring. Intravenous: sterile, endotoxin-free, hemocompatibility tests, saline/iron controls.

Note: Interpret size- and surface-dependent effects within a single, well-defined MNPs class under matched media and exposure conditions to avoid misleading cross-type comparisons. “Typical direction” summarizes trends reported across the cited examples and may vary by model and dose. Abbreviations: ^1^ BSA—bovine serum albumin; ^2^ PEG—poly (ethylene) glycol; ^3^ DSPE—1,2-disteaoryl-sn-glycero-3-phosphoethanolamine; ^4^ HA—hyaluronan; ^5^ PLGA—poly (lactic-co-glycolic acid; ^6^ DEX—dextran; ^7^ PVA—poly (vinyl alcohol); ^8^ APTMS—(3-aminopropyl)trimethoxysilane; ^9^ TEOS—tetraethyl orthosilicate; ^10^ HEC—hydroxyethyl cellulose; ^11^ PVP—poly (vinylpyrrolidone); ^12^ NCC—nanocrystalline cellulose; ^13^ CR—Congo Red; ^14^ PAA—poly (arabic acid) [poly (acrylic acid)]; ^15^ Dh—hydrodynamic diameter; ^16^ PDI—polydispersity index; ^17^ MW—molecular weight.

**Table 3:** A summary of the main in vitro toxicity tests for magnetic nanoparticles.

Purpose	Toxicity Test/Principle of the Method	Ref
Cells proliferation/viability investigation	**Colorimetric tetrazolium salts: MTT ^1^, XTT ^2^, MTS ^3^, and WSTs (i.a., WST-1 ^4^) assays**. In contrast to dead cells, tetrazolium salts are reduced in viable, metabolically active cells to intensely colored formazan products.	[83,94]
	**LDH ^5^ assay.** The enzyme is released from the cytoplasm into the cell culture medium following loss of membrane integrity, serving as an indicator of apoptosis, necrosis, or other forms of cellular damage.	[44,83,95]
	**NRU ^6^ colorimetric assay.** Viable cells incorporate neutral red into lysosomes, whereas dead cells fail to do so. The bound dye is subsequently extracted from the cytoplasm for quantification.	[96]
	**Trypan blue stain assay** (dye exclusion assay). Non-viable cells take up the dye and appear blue under light microscopy, whereas viable cells exclude it.	[90]
	**Almar blue (resazurin) assay:** A fluorometric method based on the enzymatic reduction of resazurin by viable cells into resorufin, a pink, fluorescent product that diffuses into the culture medium.	[97]
	**Inclusion dyes such as calcein-AM ^7^ and 5-CFDA-**AM ^8^ require intracellular enzymatic activity and membrane integrity. In viable cells, esterases cleave non-fluorescent precursors, producing fluorescent compounds retained within the cytoplasm.	[86]
Oxidative stress assessment	**ROS ^9^ production by DCFDA ^10^ assay.** DCFDA diffuses into the cells and is deacylated by esterases to a non-fluorescent compound, which is oxidized by ROS into the highly fluorescent DCF ^11^.	[44,99]
	**Measurement of GPx ^12^, SOD ^13^, and catalase levels.** The enzymes convert superoxide radicals into H_2_O_2_ and O_2_ (SOD), H_2_O_2_ into water (GPx), or H_2_O_2_ into O_2_ and water (catalase). The antioxidant protein expression levels can be detected using immunohistochemistry, immunofluorescence, or immunogold labeling.	[100]
	**Estimation of NO level.** Direct methods include EPR/ESR ^14^ and electrochemical assays, while indirect methods involve the determination of nitrate and nitrite concentrations.	[85,101]
	**Measurement of disruption of mitochondrial functions.** Such dysfunctions include changes in the mitochondrial membrane potential (MMP), which can be assessed by monitoring the uptake of rhodamine 123.	[102,103]
Genotoxicity evaluation	**Comet assay.** The negatively charged, low-molecular-weight DNA fragments generated by damage migrate towards the anode during electrophoresis, producing a comet-like structure. The measurement of its “tail” length and intensity reflects the extent of DNA damage.	[104]
	**8-OHdG ^15^ as a biomarker for oxidative DNA damage.** ELISA or HPLC are commonly employed to detect 8-OHdG at oxidized DNA sites, predominantly within guanine bases.	[105]
	**PI ^16^ or Hoechst dyes.** PI stains DNA and RNA in non-viable cells or cells with reversibly damaged membranes. Hoechst dyes, such as bisbenzimide (Hoechst 33342) trihydrochloride, bind to the minor groove of double-stranded DNA—preferentially at A/T-rich regions—resulting in high fluorescence intensity.	[106,107]
	**TUNEL assay.** Detects both single- and double-stranded DNA breaks by enzymatic incorporation of modified nucleotides at sites of damage.	[108]
Apoptosis detection	**Measurement of the Caspase-3 activity**. Since caspase-3 is a key executioner protease in apoptosis, its activity can be quantified using pro-fluorescent peptide substrates. Upon caspase-mediated cleavage, the fluorophore is released from the peptide backbone, yielding a measurable fluorescence signal.	[110]
	**AO/EB ^17^ staining.** Acridine orange (AO) readily diffuses into viable cells, whereas ethidium bromide (EB) penetrates only cells with compromised membranes, as in apoptosis or necrosis. Cells stained solely with AO fluoresce green, while apoptotic or membrane-compromised cells preferentially incorporate EB, yielding red fluorescence.	[84]
Immunotoxicity assessment	**LPA ^18^ assay.** LPA measures the ability of lymphocytes placed in a short-time tissue culture to undergo clonal proliferation when stimulated in vitro by an antigen, mitogen, or foreign molecules such as MNPs.	[111]
Membrane function assessment	**ATPase assay.** The ATPase assay determines the activity of membrane-bound ATPases based on the enzymatic hydrolysis of ATP to ADP and inorganic phosphate (Pi). The amount of Pi released is quantified colorimetrically, providing an indicator of membrane integrity and ion transport efficiency.	[112,113]

^1^ MTT: (3-[4,5-dimethylthiazol-2-yl]-2,5 diphenyl tetrazolium bromide); ^2^ XTT: 2,3-bis-(2-methoxy-4-nitro-5-sulfophenyl)-2H-tetrazolium-5-carboxanilide, ^3^ MTS: 3-(4,5-dimethylthiazol-2-yl)-5-(3-carboxymethoxyphenyl)-2-(4-sulfophenyl)-2H-tetrazolium; ^4^ WST-1: 2-(4-iodophenyl)-3-(4-nitrophenyl)-5-(2,4-disulfophenyl)-2H tetrazolium; ^5^ LDH: lactase dehydrogenase; ^6^ NRU: neutral red uptake; ^7^ AM: acetoxymethylester; ^8^ 5-CFDA-AM: 5-Carboxyfluorescein diacetate acetoxymethyl ester; ^9^ ROS: reactive oxygen species; ^10^ DCFDA: 2,7–dichlorofluorescin diacetate; ^11^ DCF: 2,7–dichlorofluorescein; ^12^ GPx: glutathione peroxidase, ^13^ SOD: superoxidase dismutase; ^14^ EPR/ESR: electron paramagnetic resonance spectroscopy; ^15^ 8-OHdG: 8-hydroxy-2′-deoxyguanosine; ^16^ PI: propidium iodide; ^17^ EB/AO: ethidium bromide/acridine orange; ^18^ LPA: lymphocyte proliferation assay.

## Data Availability

Not applicable.

## Abbreviations

5-CFDA-AM	5-Carboxyfluorescein Diacetate Acetoxymethyl Ester (fluorescent viability dye activated by intracellular esterases)
8-OHdG	8-Hydroxy-2′-deoxyguanosine (oxidative DNA damage biomarker)
8-dG	8-deoxyguanosine (oxidative DNA lesion, precursor form measured in DNA damage studies)
A/G	Albumin/Globulin ratio (serum protein ratio)
AEAPS	N-(2-aminoethyl)-3-aminopropyl trimethoxysilane (surface coating agent for nanoparticles)
ALB	Albumin (major serum protein)
ALP	Alkaline Phosphatase (enzyme, liver/bone function marker)
ALT	Alanine Aminotransferase (hepatic enzyme, liver function marker)
AO/EB	Acridine Orange/Ethidium Bromide (dual-staining method to distinguish viable from apoptotic/necrotic cells)
APTMS	(3-Aminopropyl)trimethoxysilane (silane coupling agent introducing amine groups)
AST	Aspartate Aminotransferase (hepatic enzyme, liver function marker)
ATP	Adenosine Triphosphate (primary energy carrier in cells)
BMP6	Bone Morphogenetic Protein 6 (regulator of systemic iron metabolism)
BSA	Bovine Serum Albumin (protein used as nanoparticle coating)
BUN	Blood Urea Nitrogen (biochemical marker of kidney function)
CAM assay	Chorioallantoic Membrane assay (in ovo method for testing angiogenesis, toxicity, and nanoparticle effects)
CCK-8	Cell Counting Kit-8 (commercial assay kit using WST-8 for cell viability measurement)
CD11b	Cluster of Differentiation 11b (integrin alpha M chain, expressed on monocytes, macrophages, NK cells, granulocytes)
CD14	Cluster of Differentiation 14 (co-receptor for bacterial lipopolysaccharide, monocyte/macrophage marker)
CD4^+^	Cluster of Differentiation 4 positive (marker of helper T lymphocytes)
CD86	Cluster of Differentiation 86 (co-stimulatory molecule expressed on antigen-presenting cells, involved in T-cell activation)
CRE	Creatinine (biochemical marker of kidney function)
Calcein-AM	Calcein Acetoxymethyl Ester (cell-permeant, non-fluorescent dye converted by esterases to fluorescent calcein in viable cells)
Ca^2+^-ATPase	Calcium Adenosine Triphosphatase (ATP-dependent calcium pump in membranes)
DBIL	Direct Bilirubin (conjugated bilirubin marker)
DCF	2′,7′-Dichlorofluorescein (fluorescent oxidation product used as ROS indicator)
DCF assay	Dichlorofluorescein assay (fluorometric method for measuring intracellular ROS generation)
DCFDA	2′,7′-Dichlorofluorescin Diacetate (fluorescent probe for intracellular ROS, also called H_2_DCFDA, DCFH-DA, or DCFH)
DCFH-DA/DCFH	2′,7′-Dichlorodihydrofluorescein Diacetate (alternative abbreviations for DCFDA)
DEX	Dextran (polysaccharide used as nanoparticle coating)
DLP	Digital Light Processing (light-based 3D bioprinting method using projected light for layer curing)
DMSA	Dimercaptosuccinic Acid (chelating agent used for nanoparticle coating)
DMSO	Dimethyl Sulfoxide (solvent used to dissolve formazan products in MTT assay)
DMT1	Divalent Metal Transporter 1 (iron transporter protein)
DPPC	1,2-Dipalmitoyl-sn-glycero-3-phosphocholine (major phospholipid component of cell membranes; model bilayer system)
DSPE-PEG	1,2-Distearoyl-sn-glycero-3-phosphoethanolamine-Polyethylene Glycol (lipid–PEG conjugate for nanoparticle coating)
Dh	Hydrodynamic diameter (effective particle size in suspension)
ECM	Extracellular Matrix (network of proteins and polysaccharides that provides structural and biochemical support to cells)
EDD	Embryonic Development Day (day of embryo development, used to standardize in ovo experiments)
ELISA	Enzyme-Linked Immunosorbent Assay (plate reader often used for colorimetric absorbance measurement)
EMA	European Medicines Agency
EPR/ESR	Electron Paramagnetic Resonance/Electron Spin Resonance (spectroscopic methods for detecting free radicals and NO)
ESR	Erythrocyte Sedimentation Rate (non-specific marker of inflammation)
EURL ECVAM	European Union Reference Laboratory for Alternatives to Animal Testing/European Centre for the Validation of Alternative Methods
FACS	Fluorescence-Activated Cell Sorting (method for multiparametric single-cell analysis and sorting)
FDA	U.S. Food and Drug Administration
FITC	Fluorescein Isothiocyanate (fluorescent dye commonly used for labeling biomolecules, here dextran-coated MNPs)
FMR	Ferromagnetic Resonance (spectroscopic technique for detecting magnetic nanoparticle distribution in tissues)
G6PD	Glucose-6-Phosphate Dehydrogenase (enzyme supporting antioxidant defenses via NADPH regeneration)
GGT	Gamma-Glutamyl Transferase (enzyme, liver function marker)
GLB	Globulin (serum protein fraction)
GMNPs	GoldMag Nanoparticles (superparamagnetic core/shell nanoparticles with Fe_3_O_4_ core and gold shell)
GPx	Glutathione Peroxidase (antioxidant enzyme)
GR	Glutathione Reductase (enzyme regenerating reduced glutathione)
GelMA	Gelatin Methacryloyl (biocompatible hydrogel commonly used as bioink in tissue engineering)
H&E	Hematoxylin and Eosin staining (routine histological staining method)
HA	Hyaluronic Acid (glycosaminoglycan used for nanoparticle coating/targeting)
HEC	Hydroxyethyl Cellulose (cationic biopolymer used for nanoparticle functionalization)
HPLC	High-Performance Liquid Chromatography (analytical separation technique)
H_2_DCFDA	2′,7′-Dichlorodihydrofluorescein Diacetate (reduced form of DCFDA)
IFC	Imaging Flow Cytometry (hybrid technique combining flow cytometry with high-resolution microscopy for single-cell imaging and quantitative analysis)
IL-6	Interleukin-6
INT	2-(4-Iodophenyl)-3-(4-nitrophenyl)-5-phenyl-2H-tetrazolium (iodonitrotetrazolium, tetrazolium salt used in LDH assays)
ISC machinery	Iron–Sulfur Cluster assembly machinery (system for Fe–S cluster biogenesis)
LDH	Lactate Dehydrogenase (enzyme, cytotoxicity/necrosis marker)
LD_50_	Median Lethal Dose (dose required to kill 50% of a test population)
LIP	Labile Iron Pool (cytosolic pool of weakly bound iron ions)
LPA	Lymphocyte Proliferation Assay (in vitro immunotoxicity test measuring clonal expansion of lymphocytes)
MMP	Mitochondrial Membrane Potential (indicator of mitochondrial integrity and function)
MNP(s)	Magnetic Nanoparticle(s)
MPI	Magnetic Particle Imaging
MPT	Mitochondrial Permeability Transition (process involving opening of the mitochondrial permeability transition pore)
MRI	Magnetic Resonance Imaging
MTS	3-(4,5-Dimethylthiazol-2-yl)-5-(3-carboxymethoxyphenyl)-2-(4-sulfophenyl)-2H-tetrazolium (tetrazolium compound used in colorimetric assays)
MTT	3-(4,5-Dimethylthiazol-2-yl)-2,5-diphenyltetrazolium bromide (tetrazolium salt used in cell viability assays)
Mg^2+^-ATPase	Magnesium Adenosine Triphosphatase (ATP-dependent magnesium transport enzyme)
MitoSOX™ Red (Mito-HE)	Mitochondria-targeted hydroethidine probe (dihydroethidium derivative conjugated with triphenylphosphonium cation, specific for mitochondrial superoxide detection)
NADH	Nicotinamide Adenine Dinucleotide (reduced form; electron donor in metabolic reactions)
NADPH	Nicotinamide Adenine Dinucleotide Phosphate (reduced form; cofactor in biosynthetic reactions)
NAD^+^	Nicotinamide Adenine Dinucleotide (oxidized form)
NAM(s)	New Approach Methodologies
NCC	Nanocrystalline Cellulose (anionic biopolymer, nanoparticle coating material)
NCOA4	Nuclear Receptor Coactivator 4 (mediator of ferritinophagy)
NRU	Neutral Red Uptake assay (colorimetric cytotoxicity assay measuring lysosomal activity)
Na^+^/K^+^-ATPase	Sodium-Potassium Adenosine Triphosphatase (membrane ion pump maintaining Na^+^/K^+^ gradients)
OECD	Organization for Economic Co-operation and Development
PDI	Polydispersity Index (measure of particle size distribution uniformity, obtained by DLS)
PEG	Polyethylene Glycol (polyether polymer used for nanoparticle surface modification)
PEI	Polyethylenimine (cationic polymer used for nanoparticle coating/transfection)
PES	Phenazine Ethosulfate (alternative electron coupling reagent in tetrazolium assays)
PET	Positron Emission Tomography
PI	Propidium Iodide (fluorescent dye binding nucleic acids in non-viable cells)
PLGA	Poly(lactic-co-glycolic acid) (biodegradable copolymer used for nanoparticle coatings)
PMN	Polymorphonuclear leukocytes (granulocytes, a subtype of white blood cells)
PMS	Phenazine Methosulfate (electron coupling reagent in tetrazolium assays)
PT pore	Permeability Transition pore (non-specific mitochondrial pore enabling solute passage < 1500 Da)
PVA	Poly(vinyl alcohol) (synthetic polymer used as nanoparticle coating)
PVP	Poly(vinylpyrrolidone) (synthetic polymer used as stabilizer and nanoparticle coating)
Pi	Inorganic Phosphate (direct product of ATP hydrolysis, measured colorimetrically in ATPase activity assays)
RBCs	Red Blood Cells (erythrocytes)
RNA-seq	RNA sequencing (high-throughput transcriptomic profiling technique)
RNS	Reactive Nitrogen Species
ROS	Reactive Oxygen Species
RT-CES	Real-Time Cell Electronic Sensing (impedance-based cytotoxicity assay)
SCGE	Single-Cell Gel Electrophoresis (formal name of the comet assay, DNA damage detection method)
SEM	Scanning Electron Microscopy
SIM	Structured Illumination Microscopy (super-resolution fluorescence microscopy technique)
SLA	Stereolithography (light-based 3D bioprinting method using photopolymerization)
SMAD	SMAD family proteins (signal transducers in BMP/TGF-β pathways)
SMART	Somatic Mutation and Recombination Test
SOD	Superoxide Dismutase (antioxidant enzyme)
SOPs	Standard Operating Procedures (standardized, detailed instructions ensuring reproducibility and consistency in experimental protocols)
SPECT	Single Photon Emission Computed Tomography
STEAP3	Six-Transmembrane Epithelial Antigen of Prostate 3 (lysosomal ferrireductase enzyme)
TBIL	Total Bilirubin (serum bilirubin marker)
TEM	Transmission Electron Microscopy
TEOS	Tetraethyl Orthosilicate (silica precursor used for hydroxyl surface functionalization)
TP	Total Protein (serum protein marker)
TRPML1/MCOLN1	Transient Receptor Potential Mucolipin 1 (endolysosomal ion channel protein)
TUNEL	Terminal deoxynucleotidyl transferase dUTP Nick End Labeling (assay for detecting single- and double-stranded DNA breaks)
UA	Uric Acid (serum biochemical marker)
WBC	White Blood Cell count
WST	Water-Soluble Tetrazolium (family of tetrazolium salts used in viability assays)
WST-1	2-(4-Iodophenyl)-3-(4-nitrophenyl)-5-(2,4-disulfophenyl)-2H-tetrazolium (mitochondrial activity-based viability assay reagent)
WST-8	2-(2-Methoxy-4-nitrophenyl)-3-(4-nitrophenyl)-5-(2,4-disulfophenyl)-2H-tetrazolium (high-sensitivity tetrazolium salt for viability assays)
XTT	2,3-Bis-(2-methoxy-4-nitro-5-sulfophenyl)-2H-tetrazolium-5-carboxanilide (water-soluble tetrazolium salt used for viability assays)
cGMP	current Good Manufacturing Practice
qRT-PCR	Quantitative Reverse Transcription Polymerase Chain Reaction (technique for quantifying mRNA expression)
^3^H-thymidine	Tritiated Thymidine (radioactive thymidine analog used to measure DNA synthesis and cell proliferation/apoptosis)
ζ (zeta potential)	Zeta Potential (indicator of surface charge and colloidal stability of nanoparticles)
**Cell Lines and Biological Models**	
A172	Human glioblastoma cell line, model for brain tumor research and drug sensitivity testing
A549	Human lung adenocarcinoma epithelial cell line
AdSCs	Adult Stem Cells (tissue-specific stem cells with limited differentiation potential)
Balb/c mice	Inbred mouse strain, frequently used in immunological and toxicological studies
C57BL/6 mice	Inbred mouse strain, most commonly used model organism in immunology, oncology, and metabolism research
CE	Chicken Embryo (experimental model used in in ovo studies with CAM)
DCs (monocyte-derived dendritic cells)	Human dendritic cells differentiated from monocytes, model for immune function and immunotoxicity
Fibrosarcoma cells	Human fibrosarcoma cell line (malignant connective tissue–derived)
HEK293	Human embryonic kidney cell line, widely used as a model for transfection, gene expression, and toxicology assays
HeLa	Human cervical cancer cell line, classical model in oncology, virology, and cytotoxicity testing
Hep3B	Human hepatocellular carcinoma cell line
HepG2	Human hepatocellular carcinoma cell line, model for hepatocyte metabolism, drug toxicity, and genotoxicity studies
hFOB	Human Fetal Osteoblast (cell line derived from human fetal bone tissue)
Human aortic endothelial cells	Primary human endothelial cells derived from the aorta
Human coronary artery endothelial cells	Primary human endothelial cells derived from coronary artery
Human monocyte-derived dendritic cells	Dendritic cells differentiated in vitro from primary human monocytes
Human skin fibroblasts	Primary or immortalized fibroblast cells derived from human skin
Human umbilical vein endothelial cells (HUVECs)	Primary human endothelial cells derived from umbilical vein
HUVECs	Human Umbilical Vein Endothelial Cells
L929	Mouse fibroblast cell line, widely used in cytotoxicity and biocompatibility assays
LNCaP	Human prostate cancer cell line (lymph node metastasis of prostate adenocarcinoma), androgen-sensitive model
MCF-7	Human breast cancer adenocarcinoma cell line, estrogen receptor–positive model for breast cancer studies
NIH3T3	Mouse embryonic fibroblast cell line, commonly used as a model for fibroblast proliferation and transformation
Normal fibroblasts	Primary or immortalized fibroblast cells (normal human connective tissue–derived fibroblasts)
Normal fibroblasts	Human fibroblast cell line (non-transformed connective tissue cells)
Pancreatic islet organoids	3D in vitro organoid model derived from pancreatic islet cells, used to study insulin secretion and metabolic function
Patient-derived pancreatic ductal adenocarcinoma organoids	Organoids cultured from human pancreatic ductal adenocarcinoma tissue, preserving tumor-specific features
Primary rat hepatocytes	Primary liver parenchymal cells isolated from rat
PSCs	Pluripotent Stem Cells (stem cells capable of differentiating into all three germ layers: endoderm, mesoderm, ectoderm)
SH-SY5Y	Human neuroblastoma cell line, model for neuronal differentiation and neurotoxicity
SHR (Spontaneously Hypertensive Rats)	Rat model of essential hypertension
SK-Hep-1	Human liver adenocarcinoma cell line (often used as hepatoma model)
THP-1	Human monocytic leukemia cell line, widely used to study monocyte/macrophage biology, immune activation, and nanoparticle uptake
U251	Human glioblastoma cell line
WKY (Wistar–Kyoto) rats	Normotensive rat strain, standard control in cardiovascular research
Zebrafish embryos	Widely used vertebrate model in developmental biology and nanotoxicology, transparent and genetically tractable
